# Fluorescent Microspheres as Point Sources: A Localization Study

**DOI:** 10.1371/journal.pone.0134112

**Published:** 2015-07-28

**Authors:** Jerry Chao, Taiyoon Lee, E. Sally Ward, Raimund J. Ober

**Affiliations:** 1 Department of Biomedical Engineering, Texas A&M University, College Station, Texas, United States of America; 2 Department of Molecular and Cellular Medicine, Texas A&M Health Science Center, College Station, Texas, United States of America; 3 Department of Electrical Engineering, University of Texas at Dallas, Richardson, Texas, United States of America; 4 Department of Microbial Pathogenesis and Immunology, Texas A&M Health Science Center, College Station, Texas, United States of America; University of Zurich, SWITZERLAND

## Abstract

The localization of fluorescent microspheres is often employed for drift correction and image registration in single molecule microscopy, and is commonly carried out by fitting a point spread function to the image of the given microsphere. The mismatch between the point spread function and the image of the microsphere, however, calls into question the suitability of this localization approach. To investigate this issue, we subject both simulated and experimental microsphere image data to a maximum likelihood estimator that localizes a microsphere by fitting an Airy pattern to its image, and assess the suitability of the approach by evaluating the ability of the estimator to recover the true location of the microsphere with the best possible accuracy as determined based on the Cramér-Rao lower bound. Assessing against criteria based on the standard errors of the mean and the variance for an ideal estimator of the microsphere’s location, we find that microspheres up to 100 nm in diameter can in general be localized using a fixed width Airy pattern, and that microspheres as large as 1 *μ*m in diameter can in general be localized using a floated width Airy pattern.

## Introduction

In single molecule microscopy, fluorescent microspheres are commonly used as fiducial markers for the correction of sample drift [1–4] and the registration of spatially misaligned images, such as those detected by different cameras [2] or acquired in different colors [4–7]. In these applications, the underlying idea is to localize the microspheres and use the estimated positions to determine the spatial adjustments necessary for the proper interpretation of the image data and of the results of analyses. The accurate localization of a microsphere thus plays a crucial role in the overall analysis of the image data. In localization-based super-resolution microscopy [1, 3, 4, 6, 7], for example, the quality of the sub-diffraction-limit reconstruction of subcellular structures is highly sensitive to the accuracy with which drift correction and image registration have been carried out.

The localization of a fluorescent microsphere is typically carried out by fitting a point spread function, such as a 2-dimensional Gaussian function [1, 3, 5] or an Airy pattern [2], to its image. Hence, an implicit assumption is that the microsphere used is small enough to be reasonably approximated as a point source. It is thus not uncommon to find that relatively small microspheres, no greater than 200 nm in diameter, are chosen for drift correction [1–4] and image registration [2, 4–7]. This is consistent with guidelines suggesting a microscope’s point spread function to be best approximated by the image of a microsphere whose diameter is significantly smaller than the microscope’s theoretical resolution, such as given by the Rayleigh criterion [8–10].

While the use of a point spread function to localize a small microsphere, presumably justified by the high degree of similarity between the microsphere’s image and the point spread function, is commonplace, its suitability remains unclear from the perspective of whether, given the mismatch between the data and the model that is fitted to the data, the estimator is able to recover, on average, the true location of the microsphere, and to do so with an accuracy that is comparable to the best accuracy that is theoretically attainable for the given data.

In this paper, we seek to address this question using simulated microsphere image data where the true location of the microsphere is known, and where the best possible accuracy can be determined by calculating the Cramér-Rao lower bound [11] using parameters with known values. The Cramér-Rao lower bound is a lower bound on the variance of any unbiased estimator of the microsphere’s location, and depends only on the statistical description of the image data, and not on the particular estimator. We also analyze experimental images of microspheres to test the applicability of the conclusions formed based on the analysis of the simulated data, though with the experimental data the true location of the microsphere is unknown, and the best possible accuracy can only be calculated using parameters with estimated values. In both cases, the analysis of a given microsphere consists of subjecting repeat images of the microsphere to maximum likelihood localization where an Airy pattern is fitted to the data, and applying a statistical evaluation of the mean (for simulated data only) and standard deviation of the obtained location estimates. Importantly, by carrying out analyses on fluorescent microspheres with diameters ranging from 50 nm to 1 *μ*m, particular attention is given to the dependence of our findings on the size of the microsphere.

We consider two approaches to the localization of a microsphere via the fitting of an Airy pattern. In the first approach, only the values of the positional coordinates of the Airy pattern, which we assume to provide direct estimates of the desired positional coordinates of the microsphere, are allowed to be changed by the maximum likelihood estimator. In this case, the width of the Airy pattern remains fixed to its theoretical or experimentally determined value, such that the estimator cannot broaden the Airy pattern to try to match the larger width of the microsphere. The Airy pattern thus truly represents the image of an in-focus point source in this approach, which one would expect to work well only when the microsphere to be localized is relatively small. In the second approach, the width of the Airy pattern can be changed by the estimator along with the positional coordinates. The estimator can therefore attempt to match the larger width of the microsphere by broadening the Airy pattern, and one would expect this approach to be more suitable for localizing microspheres of larger sizes. In this case, the broadened Airy pattern is no longer representative of the image of a point source for the given imaging conditions, though mathematically speaking it is still an Airy pattern.

In the evaluation of our results, the localization of a microsphere by the fitting of an Airy pattern is considered to be suitable if the performance of the estimator is comparable to the best performance that can in theory be achieved by an estimator. For a given set of repeat images of a microsphere, suitability is assessed by determining, for each positional coordinate, whether the distance between its true value and the mean of its estimates satisfies a threshold criterion based on the standard error of the mean, and analogously, whether the distance between the best possible standard deviation for its estimation and the standard deviation of its estimates satisfies a threshold criterion based on the standard error of the variance. As we wish to assess the estimator’s performance against the best possible performance, the standard errors of the mean and variance are taken to be the standard errors for an ideal estimator that recovers the true value and attains the Cramér-Rao lower bound. Furthermore, to determine reasonable threshold criteria based on the standard errors, results from point source localization, wherein there is no mismatch between the data and the fitted Airy pattern, are used as a guideline.

The remaining sections of this paper are organized as follows. We begin by presenting the various theoretical elements on which the current study relies, including the microsphere image model used in the generation of our simulated data, the Cramér-Rao lower bound-based accuracy benchmark against which the standard deviation of a set of location estimates is compared, and the standard error-based criteria for assessing the suitability of fitting an Airy pattern to a microsphere image. Details are then provided on the generation and analysis of our simulated and experimental image data sets, including a description of the maximum likelihood estimator that is used in the localization of microspheres. This is followed by the presentation and discussion of the results of the localizations carried out on our simulated and experimental data, in which we utilize the standard error-based criteria to evaluate the suitability of localizing microspheres of different sizes using an Airy pattern.

## Theory and Terminology

A mathematical model for the image of a fluorescent microsphere is employed in this paper for the generation of simulated data sets. It is also used in the calculation of the Cramér-Rao lower bound-based accuracy benchmark against which the standard deviation of the location estimates for a data set is compared. For a few simulated data sets (see the section *Effect of image pixelation*), this microsphere image model is used in the actual localization of a microsphere, wherein it is fitted, in lieu of an Airy pattern, to the image data. As part of the experimental data analysis (see S4 Text), it is also fitted to the image data for the independent estimation of non-location parameters such as photon counts. The derivation of this model is detailed in this section. In addition, the Cramér-Rao lower bound-based benchmark which uses the model is presented, along with terminology used in this paper in relation to the notion of *accuracy*. The standard error-based criteria used to assess the suitability of the fitting of an Airy pattern, which in turn make use of the accuracy benchmark, are also specified here.

We end this section by giving a brief presentation on the calculation of the Airy pattern and its associated Cramér-Rao lower bound-based accuracy benchmark. The Airy pattern is fitted not only to microsphere image data in this paper, but also to point source image data simulated using the Airy pattern itself. The fitting of an Airy pattern to data generated using the pattern itself entails no mismatch between the fitted model and the data, and the results obtained are used as a reference for establishing the standard error-based criteria for assessing the results from the fitting of an Airy pattern to microsphere image data. Similarly, the associated accuracy benchmark for the localization of a point source is used for comparison with the accuracy benchmark for the localization of a microsphere.

In what follows in this section, the subscripts *ob* and *im* are used to denote, respectively, object space and image space coordinates.

### Modeling the image of a microsphere

The fluorescence from a microsphere is due to the combined fluorescence of the individual dye molecules contained within its spherical volume. Assuming the dye molecules are distributed uniformly throughout its spherical volume, an in-focus microsphere of radius *r* may be represented by the function

$$O\left(x_{ob},y_{ob},z_{ob}\right)=\frac{3}{4\pi r^{3}},\;\;\;\left(x_{ob},y_{ob},z_{ob}\right)\in S,$$ (1)

where *S* = {(*x*, *y*, *z*) ∈ ℝ^3^∣*x* ^2^+*y* ^2^+*z* ^2^ ≤ *r* ^2^} is the set of points enclosed by a sphere of radius *r* that is centered at the origin of a 3-dimensional Cartesian coordinate system. The value $\frac{3}{4\pi r^{3}}$ is the inverse of the volume of the sphere, and is chosen so that the integration of *O* over its domain *S* yields unity. Defining the object space location of a microsphere to be given by the coordinates (*x*_0_, *y*_0_, *z*_0_) of its center, this mathematical description specifies a microsphere that is located at (*x*_0_, *y*_0_, *z*_0_) = (0, 0, 0), and is bisected by the focal plane *z*_*ob*_ = 0.

To model the image of a microsphere, we approximate the microscope as a linear shift-invariant system [12] with point spread function *PSF*_*z*_*ob*__(*x*_*im*_, *y*_*im*_), (*x*_*im*_, *y*_*im*_) ∈ ℝ^2^, where the subscript *z*_*ob*_ ∈ ℝ denotes the point spread function’s dependence on a point source’s object space axial position *z*_*ob*_. Under these assumptions, the image of the microsphere of Eq (1), captured at unit lateral magnification, is given by the convolution of the functions *O* and *PSF*_*z*_*ob*__:

$$\begin{array}{ll} q\left(x_{im},y_{im}\right) & =\int_{S}O\left(x_{ob},y_{ob},z_{ob}\right)PSF_{z_{ob}}\left(x_{im}-x_{ob},y_{im}-y_{ob}\right)\;dx_{ob}\;dy_{ob}\;dz_{ob}\\ & =\frac{3}{4\pi r^{3}}\int_{S}PSF_{z_{ob}}\left(x_{im}-x_{ob},y_{im}-y_{ob}\right)\;dx_{ob}\;dy_{ob}\;dz_{ob}, \end{array}$$ (2)

(*x*_*im*_, *y*_*im*_) ∈ ℝ^2^. Note that in the more general case of an out-of-focus microsphere located at *z*_0_ ≠ 0, the subscript of *PSF* is *z*_0_ − *z*_*ob*_ to account for the axial offset from the focal plane *z*_*ob*_ = 0. For the in-focus scenario considered here, the subscript is reduced to *z*_*ob*_ since *z*_0_ = 0, and since the symmetry of *O* about the focal plane allows the inversion of the sign of *z*_*ob*_. Provided that for any *z*_*ob*_ ∈ ℝ, the function *PSF*_*z*_*ob*__ integrates to unity over ℝ^2^, it is easily shown, by virtue of the function *O* integrating to unity over *S*, that *q* is itself a function that integrates to unity over ℝ^2^. As such, the image *q* is expressed in the form of an *image function* [13]. The specification of *q* as an image function importantly allows for the straightforward simulation of image data and calculation of the Cramér-Rao lower bound-based localization accuracy benchmark using the mathematical framework presented in [13].

In practice, an in-focus microsphere can be located anywhere in the focal plane, and is imaged at some lateral magnification greater than unity. To allow for an arbitrary position (*x*_0_, *y*_0_) ≠ (0, 0) in the focal plane and a general lateral magnification *M* > 0, the image function of Eq (2) is scaled and shifted to yield

$$\begin{array}{c} f\left(x_{im},y_{im}\right)=\frac{1}{M^{2}}q\left(\frac{x_{im}}{M}-x_{0},\frac{y_{im}}{M}-y_{0}\right)\\ =\frac{3}{4\pi r^{3}M^{2}}\;\int_{S}PSF_{z_{ob}}\left(\frac{x_{im}}{M}-x_{0}-x_{ob},\frac{y_{im}}{M}-y_{0}-y_{ob}\right)\;dx_{ob}\;dy_{ob}\;dz_{ob}\\ =\frac{3}{4\pi r^{3}M^{2}}\int_{0}^{2\pi }\int_{0}^{\pi }\int_{0}^{r}PSF_{w}\left(\frac{x_{im}}{M}-x_{0}-u,\frac{y_{im}}{M}-y_{0}-v\right)\;\xi^{2}\text{sin}\;\psi \;d\xi \;d\psi \;d\phi , \end{array}$$ (3)

where *u* = *ξ* sin *ψ* cos *ϕ*, *v* = *ξ* sin *ψ* sin *ϕ*, *w* = *ξ* cos *ψ*, and (*x*_*im*_, *y*_*im*_) ∈ ℝ^2^, and where a transformation to spherical coordinates has been applied to express the triple integral more explicitly. The function *f* gives the spatial distribution of the photons detected from the microsphere, and the factor $\frac{1}{M^{2}}$ is introduced to ensure that it integrates to unity over ℝ^2^.

Finally, to complete the modeling of a practical image, the distribution function *f* of Eq (3) is scaled up with multiplication by the mean number of photons *N*_*photon*_ detected over the detector plane (i.e., over ℝ^2^). Furthermore, since an image captured by a detector, such as a charge-coupled device (CCD) camera, is pixelated, the product of *N*_*photon*_ and *f* is integrated over 2-dimensional regions corresponding to the pixels of a detector. More precisely, a *K*-pixel image of an in-focus microsphere is modeled as the sequence (*μ*_1_, *μ*_2_, …, *μ*_*K*_), where for *k* = 1, …, *K*,

$$\mu_{k}=N_{photon}\int_{C_{k}}f\left(x_{im},y_{im}\right)\;dx_{im}\;dy_{im}$$ (4)

is the mean number of photons detected at the *k*th pixel, which occupies the region *C*_*k*_ in the detector plane.

The described model is customizable in that it may be computed with any point spread function *PSF*_*z*_*ob*__. In this paper, the point spread function used is that of Born and Wolf [14]:

$$PSF_{z_{ob}}\left(x_{im},y_{im}\right)=\frac{4\pi n_{a}^{2}}{\lambda^{2}}{\left|\int_{0}^{1}\;J_{0}\left(\frac{2\pi n_{a}}{\lambda }\sqrt{x_{im}^{2}+y_{im}^{2}}\rho \right)e^{\frac{j\pi n_{a}^{2}z_{ob}}{n\lambda }\rho^{2}}\rho d\rho \right|}^{2},$$ (5)

(*x*_*im*_,*y*_*im*_) ∈ ℝ^2^, *z*_*ob*_ ∈ ℝ, where *J*_0_ is the zeroth order Bessel function of the first kind, *n*_*a*_ is the numerical aperture of the objective lens, *n* is the refractive index of the lens immersion medium, and *λ* is the wavelength of the detected photons.

**Fig 1:**
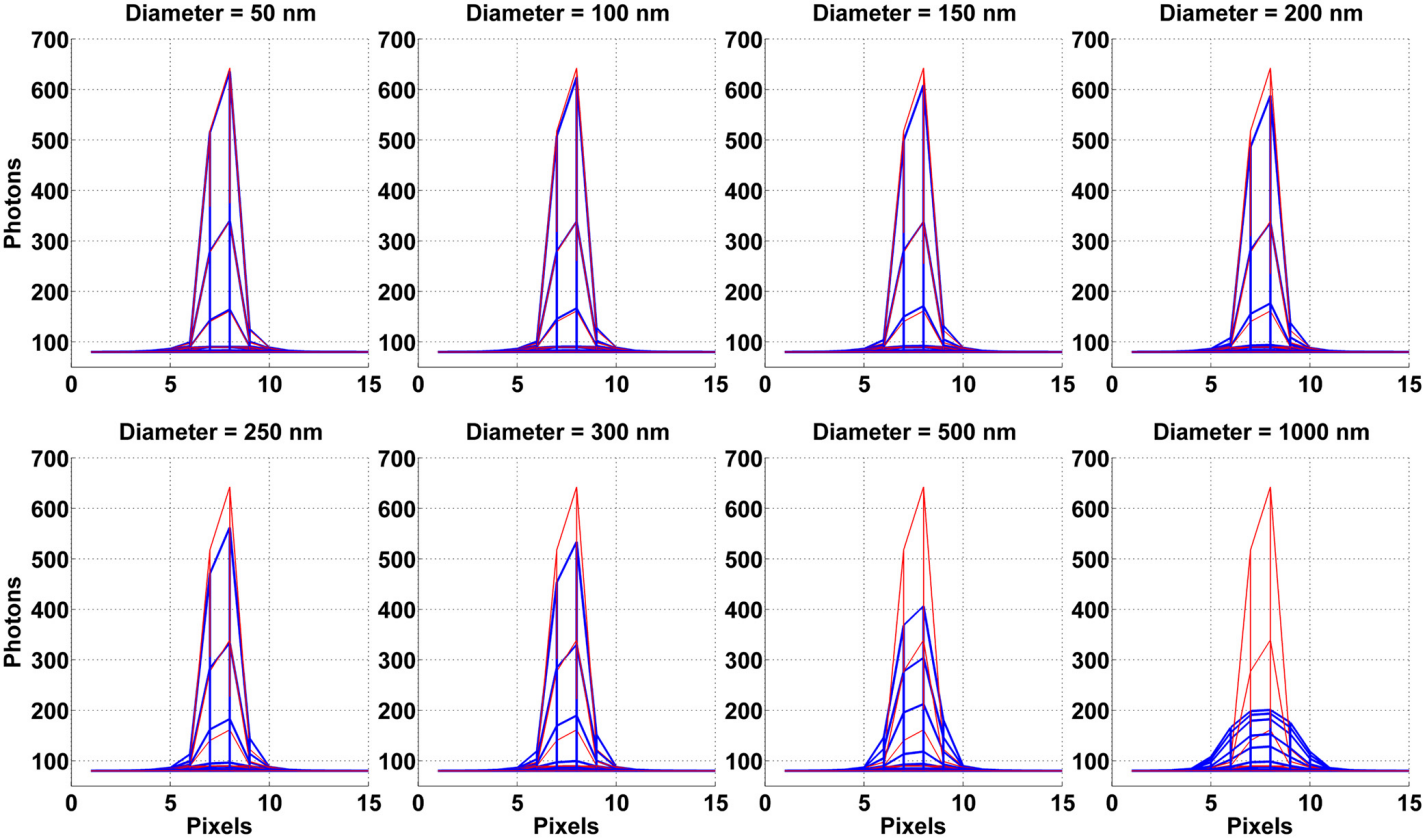
Mesh representations of images of in-focus microspheres (blue) ranging in size from 50 nm to 1 *μ*m in diameter, computed according to our model. For each size, the microsphere is assumed to emit photons of wavelength 485 nm, and to be imaged using the 63× imaging configuration specified in the section *Simulation parameters*. Each microsphere image is overlaid with the mesh representation of the Airy pattern (red) that models the image, acquired under the same conditions, of the point source that is located at the same position, and emits photons of the same wavelength, as the microsphere. In each plot, the microsphere and point source images are displayed as viewed along the *x*-axis. Values of all relevant parameters not explicitly provided here are as given in the section *Simulation parameters*.

To illustrate the image of a microsphere according to our model, Fig 1 shows the images of eight differently sized microspheres, computed using Eq (4), with diameters ranging from 50 nm to 1 *μ*m. Each image comprises a 15 × 15-pixel area, and the pixel values are plotted in a blue mesh representation viewed along the *x*-axis. As expected, it can be seen that the width of the image broadens with increasing diameter of the microsphere. Furthermore, since the mean number of microsphere photons detected over the detector plane is the same for each image, such that the 15 × 15-pixel area captures a comparable number of photons in each case, the broadening of the microsphere image is accompanied by a decrease in the height of the image.

### Localization accuracy and its limit

In this paper, both simulated and experimental images of in-focus microspheres are subjected to maximum likelihood estimation of the *x*_0_ and *y*_0_ positional coordinates of a microsphere. In a single localization analysis, estimations are carried out on repeat images of a microsphere, and standard deviations of the obtained *x*_0_ and *y*_0_ estimates are calculated. The standard deviations importantly characterize the accuracy of the estimator, and we refer to the *x*_0_ and *y*_0_ standard deviations as the *x-localization accuracy* and the *y-localization accuracy*, respectively.

For a given data set, an important component of our assessment of the suitability of localizing a microsphere by the fitting of an Airy pattern is to compare the distance between the best possible and the obtained x-localization accuracies, and the distance between the best possible and the obtained y-localization accuracies, against their respective threshold criteria based on the standard error of the variance for an ideal estimator. Since they represent accuracies that cannot be surpassed, we refer to the best possible accuracies as the *limit of the x-localization accuracy* and the *limit of the y-localization accuracy*, and denote them by *δ*_*x*_ and *δ*_*y*_, respectively. The limits of accuracy are defined to be the square root of the Cramér-Rao lower bound on the variance for estimating *x*_0_ and *y*_0_, and are therefore the best possible standard deviations for estimating *x*_0_ and *y*_0_. Denoting the vector of parameters to be estimated by *θ* = (*x*_0_, *y*_0_), *θ* ∈ Θ, where Θ is the parameter space that is an open subset of ℝ^2^, the limits of the x- and y-localization accuracy are respectively given by the expressions $\delta_{x}=\sqrt{{\left[{\text{I}}^{-1}(\theta )\right]}_{11}}$ and $\delta_{y}=\sqrt{{\left[{\text{I}}^{-1}(\theta )\right]}_{22}}$, where [**I** ^−1^(*θ*)]_*jj*_, *j* = 1, 2, is the *j*th main diagonal element of the inverse of the 2×2 Fisher information matrix

$$I(\theta )={\sum_{k=1}^{K}\left(\frac{\partial \mu_{\theta ,k}}{\partial \theta }\right)}^{T}\frac{\partial \mu_{\theta ,k}}{\partial \theta }\;\cdot \left(\int_{\mathbb{R}}\frac{{\left(\sum_{l=1}^{\infty }\;\frac{{\left[\mu_{\theta ,k}+\beta_{k}\right]}^{l-1}e^{-(\mu_{\theta ,k}+\beta_{k})}}{(l-1)!}\cdot e^{-{\left(\frac{z-l-\eta_{k}}{\sqrt{2}\cdot \sigma_{k}}\right)}^{2}}\right)}^{2}}{2\pi \sigma_{k}^{2}\cdot p_{\theta ,k}(z)}dz-1\right),$$ (6)

where *K* is the number of pixels comprising an image from which *x*_0_ and *y*_0_ are estimated, and for *k* = 1, …, *K*, *μ*_*θ*,*k*_ is the mean number of photons at the *k*th pixel that are detected from the microsphere, and is given by Eq (4), *β*_*k*_ is the mean number of photons at the *k*th pixel that are due to the background component (i.e., detected from sources other than the microsphere), *η*_*k*_ and *σ*_*k*_ are, respectively, the mean and standard deviation of the readout noise of the camera at the *k*th pixel, and *p*_*θ*,*k*_(*z*) is the probability density of the data at the *k*th pixel, given by

$$p_{\theta ,k}\left(z\right)=\frac{1}{\sqrt{2\pi }\sigma_{k}}\overset{\infty }{\underset{l=0}{\sum }}\frac{{\left[\mu_{\theta ,k}+\beta_{k}\right]}^{l}e^{-\left(\mu_{\theta ,k}+\beta_{k}\right)}}{l!}e^{-\frac{1}{2}{\left(\frac{z-l-\eta_{k}}{\sigma_{k}}\right)}^{2}},\;z\in \mathbb{R}.$$ (7)

In our analyses, a uniform background component is assumed and *β*_*k*_ is set to the same constant value *β*_0_ for *k* = 1, …, *K*. Similarly, by the assumed and actual use of a CCD camera, the readout noise at each pixel has the same mean and standard deviation, and *η*_*k*_ and *σ*_*k*_ are respectively set to the constant values *η*_0_ and *σ*_0_ for *k* = 1, …, *K*. Note that the difference between *μ*_*θ*,*k*_ in Eqs (6) and (7) and *μ*_*k*_ of Eq (4) is purely notational. The subscript *θ* is used here to explicitly denote the dependence of *μ*_*k*_ on *x*_0_ and *y*_0_.

The Fisher information matrix of Eq (6) is based on the typical assumptions that photons from the microsphere and photons from the background component are detected according to independent Poisson processes, and that the noise added to each pixel during the camera’s readout process is Gaussian distributed. Indeed, the probability density function of Eq (7) corresponds to the case where the data in each pixel is taken to be the sum of a Poisson random variable (with mean *μ*_*θ*,*k*_ + *β*_*k*_) and a Gaussian random variable (with mean *η*_*k*_ and standard deviation *σ*_*k*_) [15, 16]. The matrix of Eq (6) is expressed in a high-level form which we have reported previously [13, 16], and can be used to calculate limits of accuracy for any estimation problem, provided that the parameter vector *θ* and the functions *μ*_*θ*,*k*_ and *β*_*k*_, *k* = 1, …, *K*, are accordingly defined. Here, as per our definitions of *θ*, *μ*_*θ*,*k*_, and *β*_*k*_, the expression is applied to the localization of a microsphere in the presence of a uniform background component.

**Fig 2:**
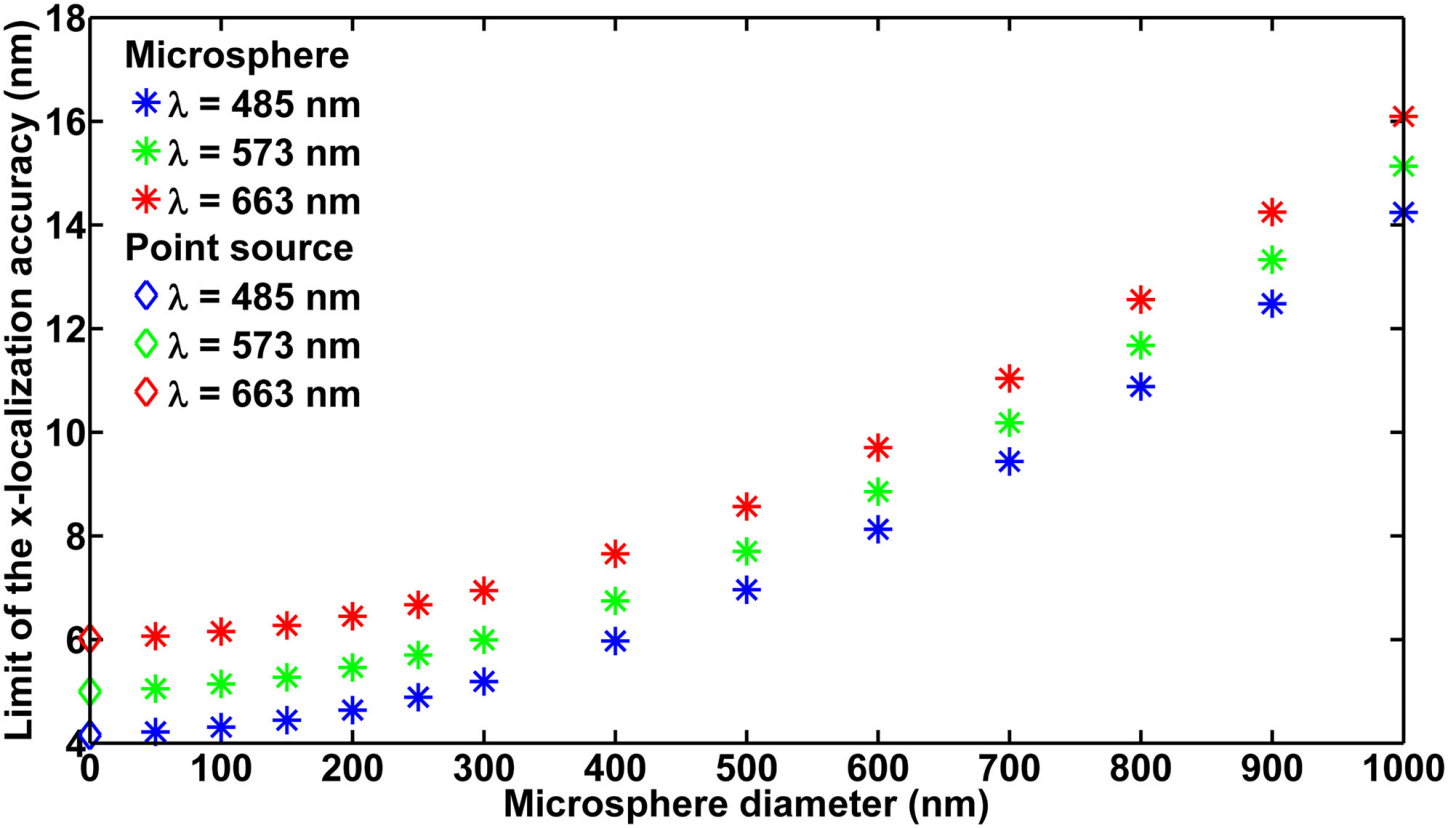
Limit of the x-localization accuracy as a function of microsphere diameter. Limits are shown for microspheres that emit photons of wavelengths 485 nm, 573 nm, and 663 nm, imaged using the 63× imaging configuration specified in the section *Simulation parameters*. Values of all parameters not explicitly provided here, including the region of interest, the location of the microsphere, and the camera readout noise standard deviation used to compute the limits, are as given in the section *Simulation parameters*. For comparison, the limit of the x-localization accuracy for the point source that is located at the same position, and emits photons of the same wavelength, as the microsphere, is shown at the diameter of 0 nm.

To give an example of limits of the localization accuracy computed as described, and to illustrate the limit’s dependence on the microsphere’s size and color, Fig 2 shows the limit of the x-localization accuracy *δ*_*x*_ as a function of the two microsphere attributes. The microsphere size ranges from 50 nm to 1 *μ*m in diameter, and the three colors considered correspond to detected photons of wavelengths 485 nm, 573 nm, and 663 nm. The figure shows that *δ*_*x*_ deteriorates with increasing microsphere size and increasing wavelength. For detailed discussions, see S1 and S2 Texts. Note that the limit of the y-localization accuracy *δ*_*y*_ as a function of microsphere size and color exhibits the same behavior and is numerically similar, and is presented in S1 Fig.

### Standard error-based threshold criteria

In the analysis of a given data set, the localization of the microsphere by the fitting of an Airy pattern is considered to be suitable if, for each positional coordinate, the distance (i.e., the absolute difference) between the true value and the mean of the estimates is within some multiple of the standard error of the mean, and the distance between the square of the limit of the localization accuracy and the variance of the estimates (i.e., the square of the localization accuracy) is within some multiple of the standard error of the variance.

So that the performance of our estimator is assessed against the best performance possible, we define the standard errors based on the performance of an ideal estimator. Specifically, the standard error of the mean is defined as

$$SE_{mean}=\frac{\sigma_{ideal}}{\sqrt{N_{sample}}}=\frac{\delta }{\sqrt{N_{sample}}},$$ (8)

where *σ*_*ideal*_ is the standard deviation of the distribution of estimates in the ideal case where an unbiased estimator attains the limit of the localization accuracy *δ*, which is either the limit of the x-localization accuracy *δ*_*x*_ or the limit of the y-localization accuracy *δ*_*y*_ described above, depending on the positional coordinate in question. The quantity *N*_*sample*_ is the sample size, given by the number of estimates from which the mean and standard deviation of the estimates are calculated for the data set. For most data sets presented in this paper, no estimates are discarded, and *N*_*sample*_ is just the number of repeat images comprising the data set. For a few data sets, a small fraction (< 5% for the majority of cases) of the pairs of *x*_0_ and *y*_0_ estimates are discarded for placing the microsphere outside the pixel array used for the localization, in which case *N*_*sample*_ is the number of pairs of *x*_0_ and *y*_0_ estimates that are retained.

The standard error of the variance, using the same notation as in Eq (8), is given by

$$SE_{var}=\sigma_{ideal}^{2}\cdot \sqrt{\frac{2}{N_{sample}-1}}=\delta^{2}\cdot \sqrt{\frac{2}{N_{sample}-1}}.$$ (9)

This formula is predicated on the distribution of estimates in the ideal case being Gaussian [17], an assumption that is justifiable by the fact that in the absence of a mismatch between the data and the fitted model, the maximum likelihood estimator, which is known to be asymptotically Gaussian distributed with variance given by the Cramér-Rao lower bound, in fact produces *x*_0_ and *y*_0_ estimates whose histograms are well fitted by a Gaussian curve with mean and standard deviation given by those of the estimates. This is the case for the results obtained from the data sets presented in the section *Effect of image pixelation* that have been simulated and fitted using the microsphere image model.

### The Airy pattern

To obtain a *K*-pixel practical image of a point source, we compute, for *k* = 1, …, *K*, *μ*_*k*_ of Eq (4) using the Airy photon spatial distribution function [13, 16]

$$f\left(x_{im},y_{im}\right)=\frac{J_{1}^{2}\left(\alpha \sqrt{{\left(\frac{x_{im}}{M}-x_{0}\right)}^{2}+{\left(\frac{y_{im}}{M}-y_{0}\right)}^{2}}\right)}{M^{2}\pi \left({\left(\frac{x_{im}}{M}-x_{0}\right)}^{2}+{\left(\frac{y_{im}}{M}-y_{0}\right)}^{2}\right)},\;\;\;\;\;\;\;\;\left(x_{im},y_{im}\right)\in {\mathbb{R}}^{2},$$ (10)

where *J*_1_ is the first order Bessel function of the first kind and $\alpha =\frac{2\pi n_{a}}{\lambda }$. As in Eq (5), *n*_*a*_ is the numerical aperture of the objective lens and *λ* is the wavelength of the detected photons. Throughout this paper, we refer to the term *α* inside the Bessel function as the *width parameter* of the Airy pattern, as its value determines how narrow or broad the Airy pattern is. The smaller its value, the broader the Airy pattern (see, e.g., S2C Fig).

To provide an illustration of the image of a point source computed in this manner, Fig 1 shows in each plot the Airy pattern, rendered as a red mesh, for the point source that is located at the same position, and emits photons of the same wavelength, as the microsphere. In this paper, we refer to such a point source as the *corresponding point source* of a given microsphere. As expected, one can see from the overlay of each microsphere image with the Airy pattern that the smaller the microsphere, the more it resembles a point source. Specifically, one observes that the microspheres with the smallest diameters of 50 nm and 100 nm have images that closely match the Airy pattern, and that the difference between the microsphere image and the Airy pattern increases with the size of the microsphere. Note that while the image of the 50-nm microsphere looks nearly identical to the Airy pattern, it is slightly shorter because despite being very small, a sphere with a 50-nm diameter is still significantly larger than a point.

To compute the limits of the x- and y-localization accuracy for the localization of a point source, the Fisher information matrix of Eq (6) is used, but with *μ*_*θ*,*k*_, *k* = 1, …, *K*, calculated for an Airy pattern using Eq (10). In addition, when the width parameter of an Airy pattern is estimated along with its positional coordinates, the limits of the localization accuracy are computed as described, but with the parameter vector *θ* = (*x*_0_, *y*_0_, *α*). Moreover, to apply the standard error-based threshold criteria to the results of point source localization, the standard error expressions in Eqs (8) and (9) are used with the limit of the localization accuracy *δ* computed as described here for an Airy pattern. Note that the assumption of Gaussian-distributed estimates for the standard error of the variance is also justifiable in the case of a point source. The *x*_0_ and *y*_0_ estimates from data sets simulated and fitted using the Airy pattern, such as those of the point source data sets presented in the section *Results and Discussion*, yield histograms that are well fitted by a Gaussian curve with mean and standard deviation given by those of the estimates.

#### Comparing limits of the localization accuracy

In Fig 1, comparison of images of microspheres with the Airy pattern of the corresponding point source shows that the smaller the microsphere, the more its image resembles an Airy pattern. This corroborates the expectation that the smallest microspheres best approximate a point source. Support for the same conclusion can be found by comparing the best possible accuracies for localizing microspheres of different sizes and the corresponding point source. For example, it can be seen in Fig 2 that the limit of the x-localization accuracy for the 50-nm microsphere is closest, and in fact very comparable, to that for the corresponding point source (plotted at diameter of 0 nm) at all three of the wavelengths considered. At the 485-nm wavelength, the difference between the two limits of accuracy, expressed as a percentage of the limit of accuracy for the point source, is only 1.46%. At the 573-nm and 663-nm wavelengths, the differences are even smaller at 1.12% and 0.77%, respectively. As the microsphere becomes larger, the limit of accuracy worsens, becoming increasingly more different from that for the corresponding point source. For the 100-nm microsphere, for example, the differences are 3.69%, 2.97%, and 2.26% at wavelengths of 485 nm, 573 nm, and 663 nm, respectively. The differences increase further to 11.59%, 9.34%, and 7.15% for the 200-nm microsphere, and to 24.87%, 19.98%, and 15.35% for the 300-nm microsphere.

## Methods

### Microsphere sample preparation

A fluorescent microsphere sample was prepared by diluting Fluoresbrite yellow green carboxylate microspheres (Polysciences, Inc., Warrington, PA) in 1X phosphate buffered saline (PBS) solution. After agitation using a vortex mixer to help minimize clumping of the microspheres, a 200-*μ*L aliquot of the sample was added to the polylysine-coated micro-well of a MatTek dish (MatTek Corporation, Ashland, MA). The dish was allowed to air-dry for 24 hours, leaving the microspheres bound to the micro-well. Before imaging, 1 mL of ultrapure filtered water was added to the dish, providing additional weight to help keep the dish from moving during imaging. Samples of 50-nm, 100-nm, 200-nm, 300-nm, 500-nm, and 1-*μ*m microspheres were prepared using this procedure.

### Experimental image acquisition

Imaging of a microsphere sample, prepared as described above, was carried out on a Zeiss Axio Observer.A1 microscope (Carl Zeiss, Thornwood, NY). The sample was imaged using a Zeiss 63×, 1.4 NA Plan-Apochromat oil immersion objective lens, with immersion oil (Immersol 518 F (Carl Zeiss)) of refractive index 1.518. A 405-nm laser (Opto Engine LLC, Midvale, UT) was reflected by a z405bcm dichroic mirror (Chroma Technology, Bellows Falls, VT) to excite the microsphere sample, and the resulting fluorescence signal collected by the objective lens was passed through an HQ480/40m CFP emission filter (Chroma Technology). The filtered fluorescence signal was captured by an ORCA-ER (C4742-95-12ER) camera (Hamamatsu Corporation, Bridgewater, NJ), which acquired images of 50-nm microsphere samples at a rate of 7.4 frames per second (exposure time of 135.0 ms per frame), and images of all other microsphere samples at a rate of 14.8 frames per second (exposure time of 67.5 ms per frame). The camera was operated in 2×2 binning mode, which translates to a pixel size of 12.9 *μ*m × 12.9 *μ*m. The size of each acquired image was 256×256 pixels. The offset of the camera was 204 digital counts, determined by computing the average value of the pixels of dark frames acquired using the same settings as for the microsphere data, but at a rate of 29.6 frames per second (exposure time of 33.8 ms per frame). This offset was subtracted from each data image prior to analysis, so that the mean of the camera readout noise was set to 0 electrons for data analysis. The standard deviation of the camera readout noise was 8.5 electrons, obtained by applying the differenced frame method (e.g., [18]) to the dark frames and multiplying the result by the gain conversion factor of 4.6 electrons per digital count. The conversion factor was taken from the camera specification and verified using a mean-variance plot analysis (e.g., [19]).

A data set consisted of 500 repeat images of an in-focus microsphere acquired using the settings specified above. Given a sample, a microsphere was manually chosen for imaging only if it was well isolated from other microspheres. This criterion helped to ensure a relatively uniform distribution of background photons over a relatively large region around the microsphere. In addition, to increase the likelihood that a chosen microsphere was truly a single microsphere and not a cluster of microspheres, it was verified to have average intensity compared with the other microspheres in the field of view. Once chosen, a close-up of the microsphere was obtained using the zoom feature of the acquisition viewer, and the focus of the microscope was manually adjusted to bring the microsphere into focus before starting the image acquisition.

### Computations

All computations, including the calculation of limits of the localization accuracy, the simulation of image data, and the maximum likelihood localization of microspheres from image data, were carried out using MATLAB (The MathWorks, Inc., Natick, MA) and functions from its Statistics and Optimization Toolboxes.

### Image simulation

In our simulations, the image detector was assumed to be a CCD camera with square pixels. A *K*-pixel image of an in-focus fluorescent microsphere or point source was simulated as a sequence of pixel values (*z*_1_, *z*_2_, …, *z*_*K*_), where for *k* = 1, …, *K*, the value *z*_*k*_ ∈ ℝ is the sum of two random numbers. The first random number represents the readout noise of the camera, and was drawn from the Gaussian distribution with mean *η*_0_ electrons and standard deviation *σ*_0_ electrons. The second random number represents the number of photons detected at the *k*th pixel, and was drawn from the Poisson distribution with mean *μ*_*k*_ + *β*_0_. The term *β*_0_ is a nonnegative constant representing the mean number of photons at each and every pixel owing to a uniform background component. The term *μ*_*k*_, given by Eq (4), represents the mean number of photons at the *k*th pixel that are detected from the microsphere or point source. In the evaluation of the integral of Eq (4), the region *C*_*k*_ was taken to be the square region corresponding to the *k*th pixel. In the case of a microsphere, the photon spatial distribution function of Eq (3) was used to compute *μ*_*k*_. In the case of a point source, *μ*_*k*_ was computed using the Airy photon spatial distribution function of Eq (10). In our implementation, the Gaussian and Poisson random numbers were respectively generated using MATLAB’s *randn* and *poissrnd* functions.

A simulated data set consisted of 1000 repeat images of a microsphere or point source, each generated (with the same Gaussian mean *η*_0_ and standard deviation *σ*_0_, and the same Poisson mean *μ*_*k*_ + *β*_0_, for *k* = 1, …, *K*) according to the procedure described.

#### Simulation parameters

We considered microspheres of thirteen different sizes, ranging from a small diameter of 50 nm to a relatively large diameter of 1 *μ*m. In combination with the different sizes, we considered three differently colored microspheres that emit fluorescence at wavelengths of *λ* = 485 nm (blue), *λ* = 573 nm (yellow green), and *λ* = 663 nm (red), so that different parts of the visible spectrum were represented in our study. We further assumed the use of two different imaging configurations to acquire the data. In the *100× imaging configuration*, it was assumed that an oil immersion objective with a magnification of *M* = 100 and a numerical aperture of *n*_*a*_ = 1.4 was used in conjunction with oil of refractive index *n* = 1.518 and a CCD camera with 16-*μ*m × 16-*μ*m pixels, and that on average, *β*_0_ = 120 background photons were detected in each pixel of an image. In the *63× imaging configuration*, it was assumed that a water dipping objective with a magnification of *M* = 63 and a numerical aperture of *n*_*a*_ = 0.9 was used in conjunction with a CCD camera with 12.9-*μ*m × 12.9-*μ*m pixels. The refractive index of water was taken to be *n* = 1.333, and it was assumed that on average, *β*_0_ = 80 background photons were detected in each pixel of an image.

In both imaging configurations, the mean and standard deviation of the CCD camera’s readout noise were set to *η*_0_ = 0 electrons and *σ*_0_ = 6 electrons, respectively. Furthermore, for each image of a microsphere, *N*_*photon*_ = 2000 photons, on average, were assumed to be detected at the detector plane. A 15×15-pixel region of interest (ROI), which captures at least 95% of those photons (precise percentage depends on the particular combination of microsphere size, emission wavelength, and imaging configuration), was simulated and used for the localization of the microsphere. The lateral location of the microsphere, which was to be estimated, was set to 7.3 pixels in the *x* direction and 7.1 pixels in the *y* direction within the 15×15-pixel ROI.

To provide a reference for interpreting the results from the analysis of microsphere data sets, we also considered point sources of the same colors and imaged using the same configurations.

Examples of model images computed using the simulation parameters can be seen in Fig 1, where the microsphere and point source images shown are computed for a wavelength of 485 nm and according to the settings for the 63× imaging configuration.

### Maximum likelihood localization

Maximum likelihood estimation was used to localize microspheres from both experimental and simulated CCD image data, as well as point sources from simulated CCD image data. More precisely, given a *K*-pixel image with pixel values (*z*_1_, *z*_2_, …, *z*_*K*_), the parameter vector *θ* = (*x*_0_, *y*_0_), or *θ* = (*x*_0_, *y*_0_, *α*) when the width of the fitted Airy pattern is allowed to be altered, was estimated by maximizing the natural logarithm of the image’s likelihood function *L*. For a single pixel, the likelihood function is simply the assumed probability density of the data at the pixel, viewed as a function of *θ*. Therefore, by the standard assumption that the data collected in the pixels comprising an image are mutually independent, the likelihood function for an image is the product of the assumed probability densities of the data in its pixels. Writing the logarithm of a product as a sum of logarithms, the function that was maximized in the localization of a microsphere or point source from a *K*-pixel image is thus given by

$$\text{ln}\left(L(\theta |z_{1},z_{2},\ldots ,z_{K})\right)\;=\;\sum_{k=1}^{K}\text{ln}\left(p_{\theta ,k}\left(z_{k}\right)\right),$$ (11)

where *p*_*θ*,*k*_, *k* = 1, …, *K*, is the assumed probability density function of the data at the *k*th pixel, given by Eq (7). In our implementation, the negative of Eq (11) was minimized using MATLAB’s *fminunc* function.

Importantly, the probability density function of Eq (7) was defined in one of two ways, through the function *μ*_*k*_ of Eq (4), to fit either an Airy pattern or the microsphere image model to the image data. For the fitting of an Airy pattern, the Airy photon spatial distribution function of Eq (10) was used to compute *μ*_*k*_. For the few examples entailing the fitting of the microsphere image model (see the section *Effect of image pixelation*), the photon spatial distribution function of Eq (3) was used to compute *μ*_*k*_.

## Results and Discussion

In this section, we assess the suitability of using an Airy pattern to localize a microsphere by interpreting the results of maximum likelihood localizations carried out on both simulated and experimental data sets generated for and under conditions typical of single molecule microscopy. We first describe the determination of, and give the rationale for, the criteria used to evaluate the suitability of our estimator. We then present and analyze our results.

### Determination of standard error-based threshold criteria

As specified in the section *Standard error-based threshold criteria*, for a given data set consisting of repeat images of a microsphere, the use of an Airy pattern for localization is determined to be suitable if, for each positional coordinate, the distance between the mean of the estimates and the true value is within some multiple of the standard error of the mean for an ideal estimator, and the distance between the variance of the estimates and the square of the limit of the localization accuracy is within some multiple of the standard error of the variance for an ideal estimator.

For both the mean and the variance, we choose the criterion to be three times their respective standard errors. This choice stems from the idea that the performance of the estimator should be comparable to the performance that can be expected when there is no mismatch between the data and the fitted model, and is therefore based on results obtained from maximum likelihood localizations where an Airy pattern is fitted to images that have been simulated with the Airy pattern itself. Such studies indicate that in the absence of a mismatch between the data and the fitted Airy pattern, the maximum likelihood estimator produces results that mostly fall within two times, but occasionally fall between two and three times the standard error for an ideal estimator. This can be seen, for example, in Tables 1 and 2, which present the results of localization carried out on six data sets, with and without the simultaneous estimation of the width of the fitted Airy pattern. The six data sets each consists of 1000 repeat images of a point source, simulated with an Airy pattern with parameters corresponding to one of the six combinations of wavelength and imaging configuration specified in the section *Simulation parameters*. From these findings, as well as the results reported in [20] suggesting that a mismatch between the data and the fitted model can lead to a significant difference between the localization accuracy and the limit of accuracy, we infer that three times the standard error makes a reasonable criterion for the fitting of an Airy pattern to microsphere image data. To give a more informative view of our results, however, we identify instances where the more stringent criterion of two times the standard error is satisfied.

**Table 1:** Maximum likelihood point source localization with a fixed width Airy pattern.

Img. Cfg.	*λ* (nm)		True value (nm)	Mean of estimates (nm)	Mean − True (nm)	*δ* (nm)	SD (nm)	(SD − *δ*)/*δ* ×100 (%)
63×	485	*x*_0_	1494.76 1453.81	1494.52	−0.24*	4.16	4.26	2.59*
		*y*_0_		1453.81	0.00*	4.32	4.12	−4.78^†^
	573	*x*_0_		1494.70	−0.06*	4.99	5.08	1.80*
		*y*_0_		1454.05	0.24*	5.19	5.32	2.59*
	663	*x*_0_		1494.64	−0.12*	6.02	6.02	0.01*
		*y*_0_		1453.88	0.07*	6.03	6.01	−0.33*
100×	485	*x*_0_	1168.00 1136.06	1168.00	−0.00*	2.99	2.98	−0.57*
		*y*_0_		1136.06	0.06*	2.74	2.63	−4.10*
	573	*x*_0_		1168.05	0.05*	3.38	3.41	0.89*
		*y*_0_		1136.11	0.11*	3.43	3.46	0.89*
	663	*x*_0_		1167.90	−0.10*	3.94	3.80	−3.45*
		*y*_0_		1136.28	0.28^†^	4.11	4.09	−0.56*

Results are shown for six data sets, each consisting of 1000 repeat images of a point source simulated with an Airy pattern with parameters corresponding to one of six combinations of wavelength *λ* and imaging configuration (see the section *Simulation parameters*). Each image in a data set was fitted with an Airy pattern whose positional coordinates *x*_0_ and *y*_0_ were estimated, but whose width parameter *α* was fixed to the value determined by the numerical aperture and wavelength. For each data set, the mean and accuracy (i.e., standard deviation SD) of the *x*_0_ and *y*_0_ estimates are shown alongside their respective true values and accuracy limits *δ*. For each coordinate, superscripts † and * for the difference (mean − true) indicate ∣mean − true∣ is between 2 and 3 times, and within 2 times, the standard error of the mean *SE*_*mean*_ for an ideal estimator, respectively. Similarly, superscripts † and * for the % difference between SD and *δ* indicate ∣SD^2^ − *δ* ^2^∣ is between 2 and 3 times, and within 2 times, the standard error of the variance *SE*_*var*_ for an ideal estimator, respectively.

**Table 2:** Maximum likelihood point source localization with a floated width Airy pattern.

Img. Cfg.	*λ* (nm)		True value (nm)	Mean of estimates (nm)	Mean − True (nm)	*δ* (nm)	SD (nm)	(SD − *δ*)/*δ* ×100(%)
63×	485	*x*_0_	1494.76 1453.81	1494.43	−0.33^†^	4.17	4.31	3.29*
		*y*_0_		1453.77	−0.04*	4.33	4.12	−4.88^†^
	573	*x*_0_		1494.69	−0.07*	5.01	5.11	2.11*
		*y*_0_		1454.03	0.22*	5.19	5.33	2.66*
	663	*x*_0_		1494.65	−0.11*	6.02	6.03	0.13*
		*y*_0_		1453.88	0.07*	6.03	6.01	−0.35*
100×	485	*x*_0_	1168.00 1136.00	1167.94	−0.06*	3.47	3.42	−1.49*
		*y*_0_		1136.05	0.05*	2.85	2.77	−2.75*
	573	*x*_0_		1167.96	−0.04*	3.43	3.48	1.62*
		*y*_0_		1136.08	0.08*	3.45	3.50	1.39*
	663	*x*_0_		1167.87	−0.13*	3.94	3.82	−3.08*
		*y*_0_		1136.25	0.25*	4.11	4.09	−0.43*

Results are shown for localization carried out on the same six data sets from Table 1, but with the width parameter *α* of the fitted Airy pattern estimated along with its positional coordinates *x*_0_ and *y*_0_. All details are as given in Table 1.

In order to draw conclusions with reference to the effect of microsphere size, our analysis of the suitability of localizing a microsphere using an Airy pattern is carried out on microspheres of different sizes. In the interpretation of our results, we take into consideration the stochastic nature of the data, in the sense that while the mean and accuracy of the estimates obtained from one set of images might satisfy their respective suitability criteria, the mean and accuracy of the estimates obtained from a different, but statistically identical set of images, might not. In other words, depending on the particular data set analyzed, different conclusions can be reached about whether an Airy pattern can be used to localize microspheres of a given size. In the case of simulated data, we therefore base our conclusions not only on the data sets that are used to drive the discussion, but also on an additional set of statistically identical data sets which produced similar results. In the case of experimental data, we base our assessment, for each microsphere size considered, on the results from five data sets, each of a different microsphere of the same size. In both cases, the conclusions presented are made conservatively by placing emphasis on results that are common to the data sets considered. For example, if the data sets on which a conclusion is based suggest different microsphere sizes as the cutoff size for localization using an Airy pattern, we lean towards the smallest of the sizes to be the cutoff.

### Simulated data

In this section, we present and discuss the results of maximum likelihood localizations carried out on simulated images of microspheres. A complete view of the simulation and analysis of the data sets is provided in S3 Text.

#### Microsphere localization using a fixed width Airy pattern

Comparison of the images of differently sized microspheres with the Airy pattern of their corresponding point source in Fig 1 suggests that the Airy pattern is most appropriate for fitting images of the smallest microspheres. Comparison using limits of the localization accuracy in Fig 2 points to the same conclusion. We report here the results of the maximum likelihood localization of microspheres from simulated data, wherein the Airy pattern for the corresponding point source is fitted to microsphere images. The results lend support to the same conclusion, but from the perspective of the ability of the estimator to recover the true location of the microsphere with the best possible standard deviation.

**Fig 3:**
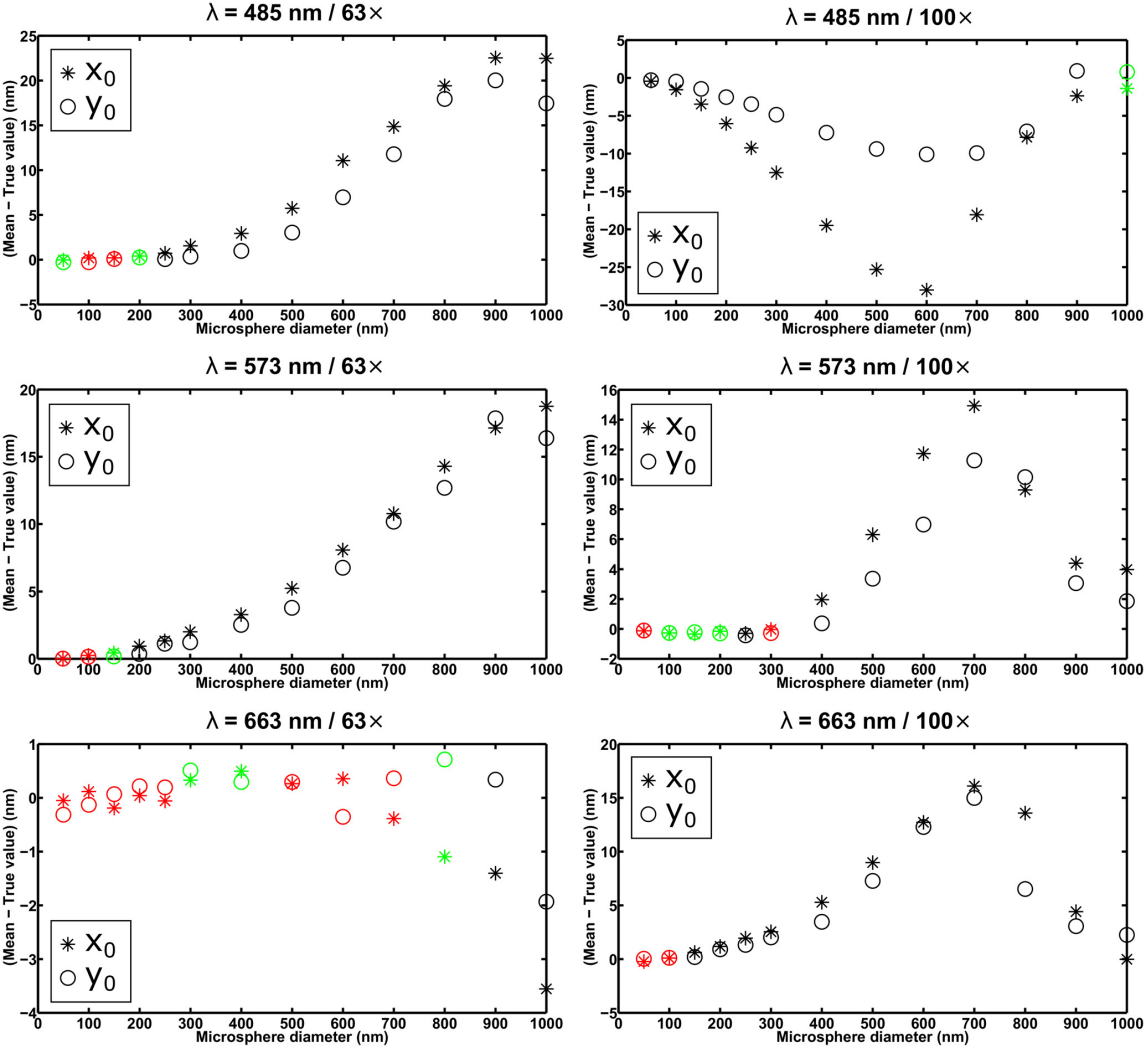
Analysis of the mean of estimates from the maximum likelihood localization of microspheres with a fixed width Airy pattern. Each plot shows the results for 13 data sets, each consisting of 1000 repeat images of a microsphere of a different size, simulated with parameters corresponding to one of six combinations of wavelength and imaging configuration (see the section *Simulation parameters*). Each image in a data set was fitted with an Airy pattern whose positional coordinates *x*_0_ and *y*_0_ were estimated, but whose width parameter *α* was fixed to the value determined by the numerical aperture and wavelength used to generate the data set. For each data set, the differences between the mean of the *x*_0_ estimates and the true value *x*_0_, and between the mean of the *y*_0_ estimates and the true value *y*_0_, are plotted in green and red if both of their magnitudes are within 3 and 2 times, respectively, their respective standard errors of the mean for an ideal estimator.

**Fig 4:**
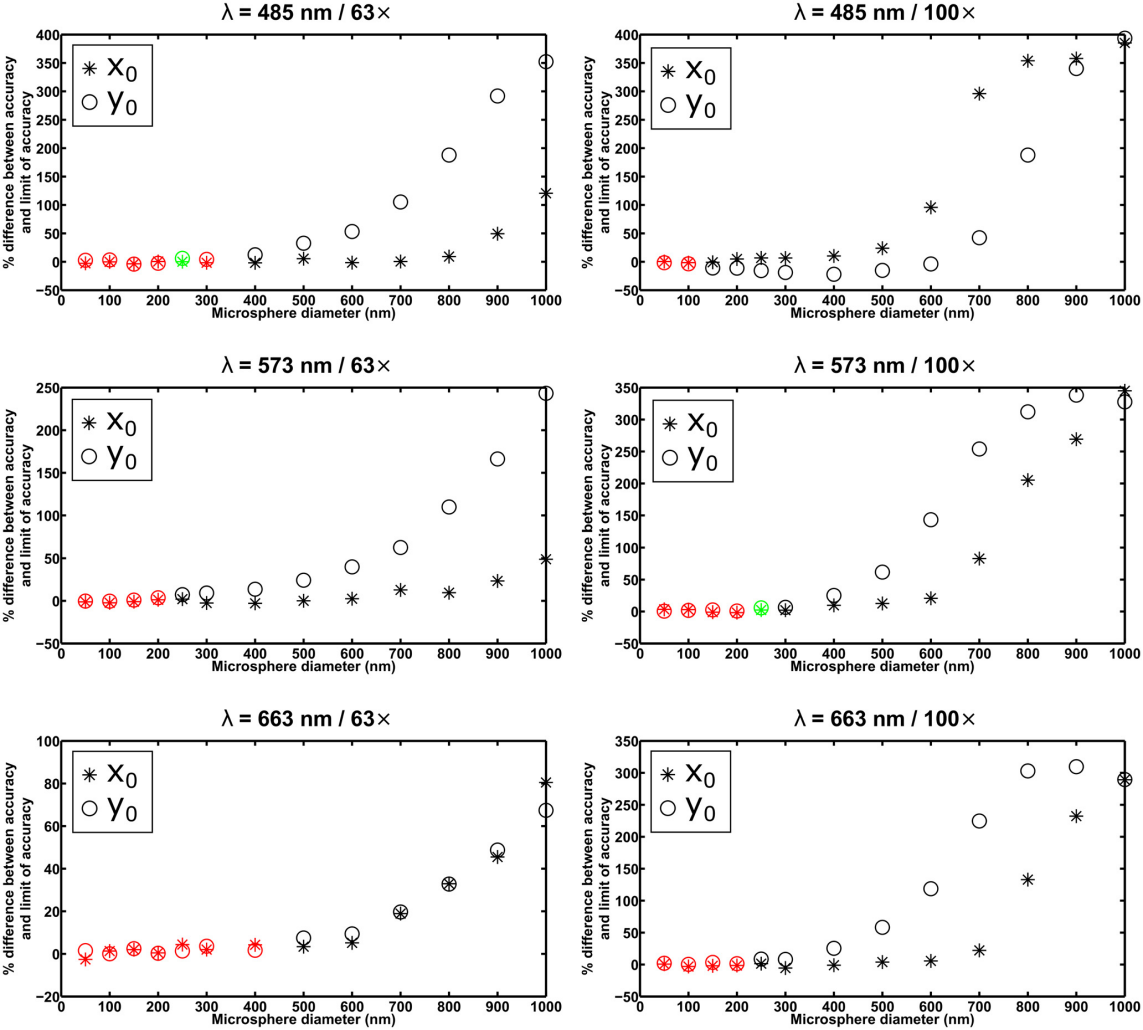
Analysis of the accuracy (i.e., the standard deviation) of estimates from the maximum likelihood localization of microspheres with a fixed width Airy pattern. The results shown are for the same *x*_0_ and *y*_0_ estimates whose averages are analyzed in Fig 3. For each data set, the percentage differences between the x-localization accuracy and the limit of the x-localization accuracy, and between the y-localization accuracy and the limit of the y-localization accuracy, are plotted in green and red if the corresponding absolute differences between the square of the localization accuracy (i.e., the variance of the estimates) and the square of the limit of accuracy are both within 3 and 2 times, respectively, their respective standard errors of the variance for an ideal estimator. The percentages are specified with respect to the limit of accuracy.

For each of the six combinations of wavelength and imaging configuration specified in the section *Simulation parameters*, Fig 3 shows, as a function of microsphere size, the difference between the mean of the *x*_0_ estimates and the true value *x*_0_, and the difference between the mean of the *y*_0_ estimates and the true value *y*_0_. For a given microsphere size, the differences are highlighted in green if both are within the criterion of three times their respective standard errors of the mean in magnitude, and in red if both are within two times their respective standard errors of the mean in magnitude. Furthermore, Fig 4 shows, for the same data sets, the percentage difference between the x-localization accuracy (i.e., the standard deviation of the *x*_0_ estimates) and the limit of the x-localization accuracy, and the percentage difference between the y-localization accuracy (i.e., the standard deviation of the *y*_0_ estimates) and the limit of the y-localization accuracy. The percentage difference is the difference between the accuracy and the limit, expressed as a percentage of the limit, and is color-coded as in the case of the difference between the mean and the true value in Fig 3, but based instead on the comparison of the standard error of the variance with the absolute difference between the square of the accuracy (i.e., the variance of the estimates) and the square of the limit.

In general, for both the mean and the accuracy of the estimates, the criterion of three times the standard error is satisfied only up to a certain microsphere size, corroborating the idea that fitting with an Airy pattern is suitable only for the localization of the smallest microspheres. The precise cutoff diameter, however, depends on the particular combination of wavelength and imaging configuration. For example, taking the smallest size that satisfies both standard error-based criteria, the plots in Figs 3 and 4 for the *λ* = 663 nm / 63× combination suggest that microspheres with a diameter of up to 400 nm can be localized with acceptable performance using an Airy pattern. The plots for the *λ* = 485 nm / 100× combination, however, suggest that even a 50-nm microsphere might be too large to be localized using an Airy pattern (see the section *Effect of image pixelation* for further discussion involving this scenario). All in all, based on the results for all six scenarios shown in Figs 3 and 4, as well as comparable results from the analysis of a second set of statistically identical data sets (see S3 and S4 Figs) we find 100 nm to be a reasonable, generally applicable rule-of-thumb cutoff diameter for fitting with an Airy pattern. This rule of thumb is further supported by similar results from the analysis of data sets simulated according to the same six combinations of wavelength and imaging configuration, but with the microsphere location changed to 7.2 pixels in the *x* direction and 7.4 pixels in the *y* direction (see S7 and S8 Figs).

Note that in Fig 3, the seemingly non-random patterns of the difference between mean and true value as a function of microsphere size appear to be examples of systematic errors due to the mismatch between the data and the fitted model, perhaps made particularly prominent by the fact that the estimator can change only the position, and not the shape, of the model. Considering that the microsphere is also positioned asymmetrically within the ROI, these errors are not unexpected.

#### Microsphere localization using a floated width Airy pattern

The above results and discussion pertain to the case where the maximum likelihood estimator is not able to alter the width of the Airy pattern that is fitted to the microsphere image data. Here, we examine the results obtained when the same simulated data sets are subjected to localizations in which the width parameter *α* of the Airy pattern is estimated simultaneously with the *x*_0_ and *y*_0_ positional coordinates. As one might expect, the results indicate that by allowing the maximum likelihood estimator to increase the width of the Airy pattern to try to match the larger width of the microsphere, this approach enables the localization of larger microspheres with acceptable performance.

**Fig 5:**
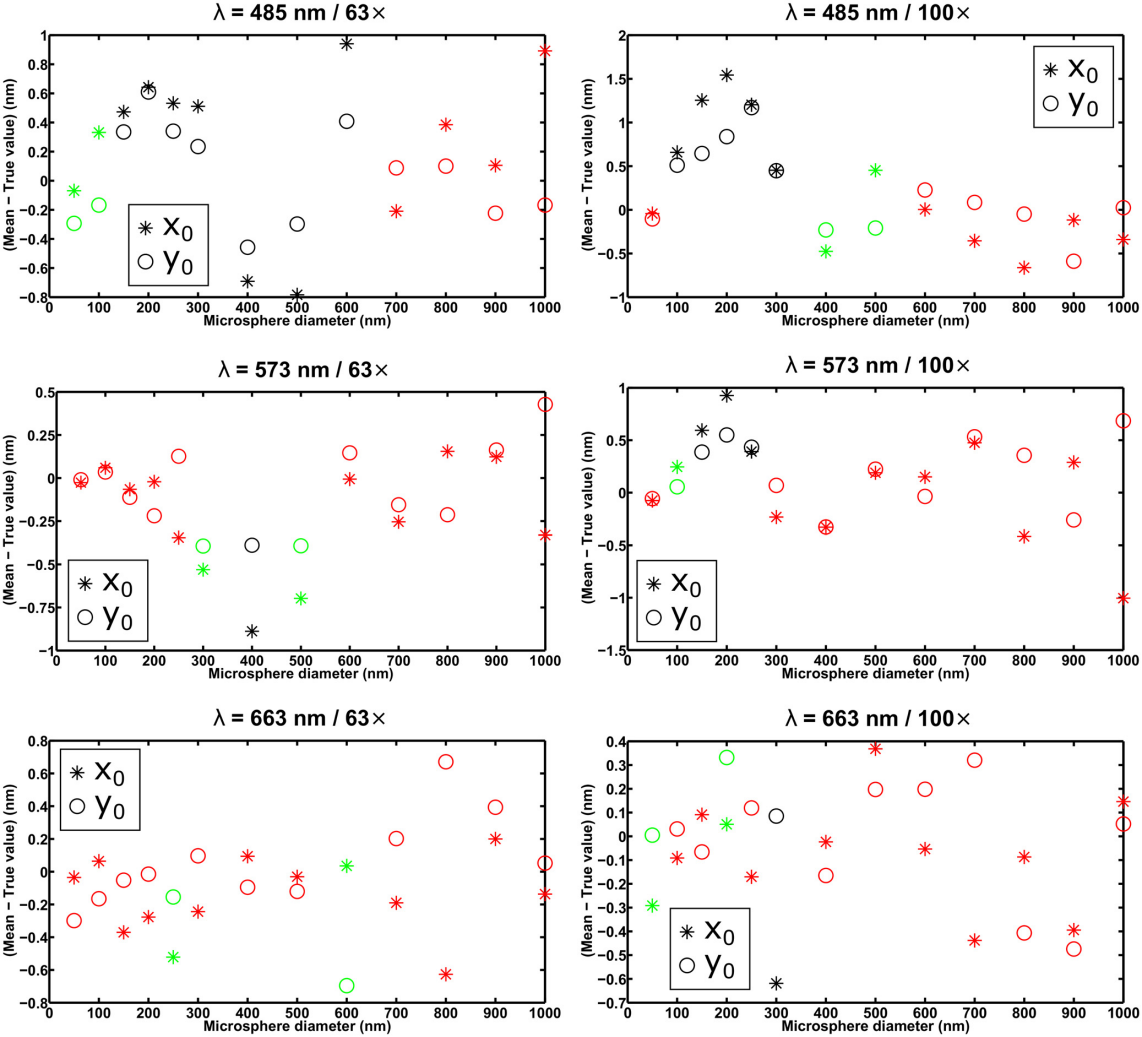
Analysis of the mean of estimates from the maximum likelihood localization of microspheres with a floated width Airy pattern. The results shown are obtained from localization carried out on the same data sets as in Fig 3, but with the width parameter of the fitted Airy pattern estimated along with its positional coordinates *x*_0_ and *y*_0_. For each data set, the differences between the mean of the *x*_0_ estimates and the true value *x*_0_, and between the mean of the *y*_0_ estimates and the true value *y*_0_, are plotted in green and red if both of their magnitudes are within 3 and 2 times, respectively, their respective standard errors of the mean for an ideal estimator.

**Fig 6:**
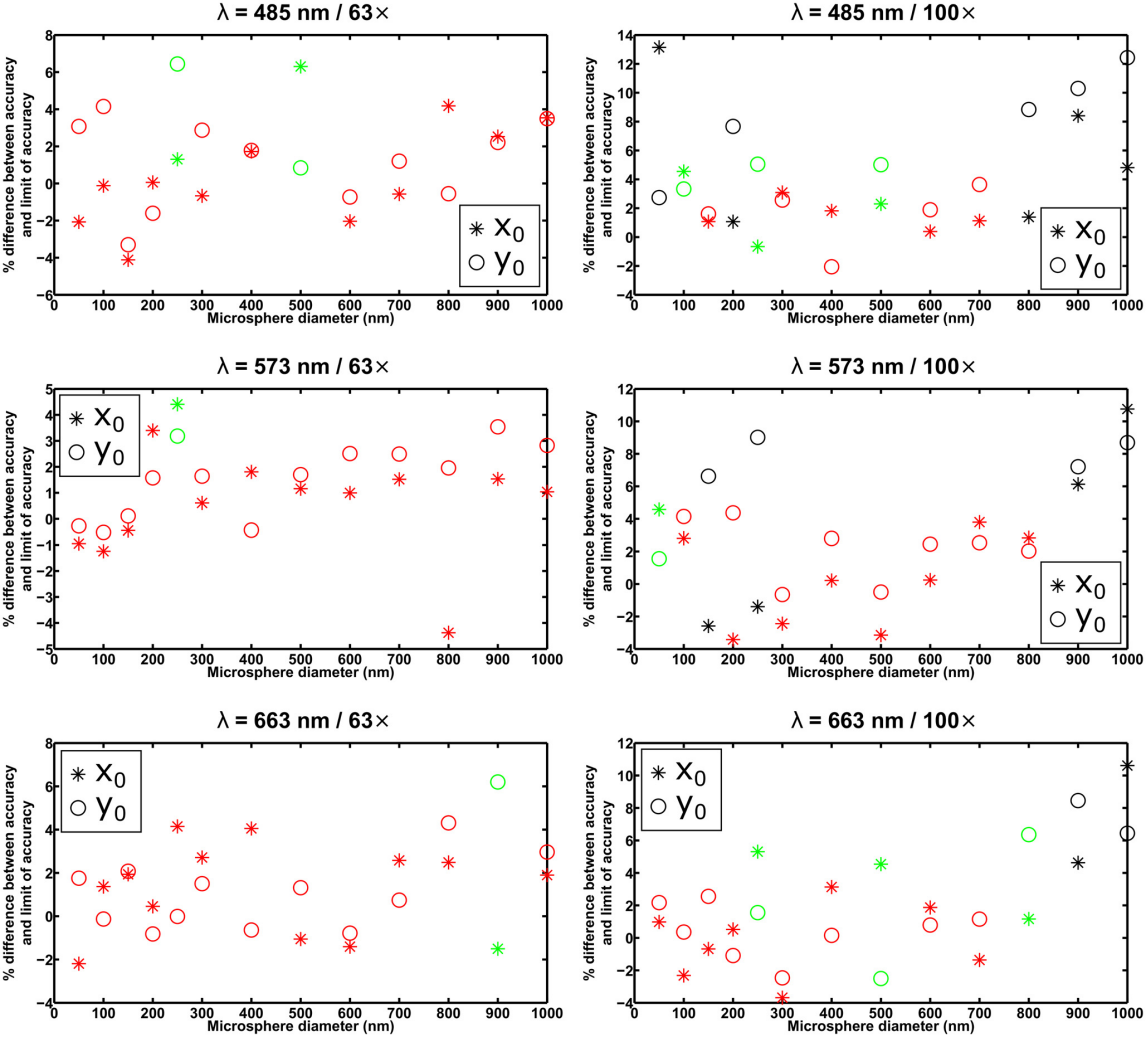
Analysis of the accuracy (i.e., the standard deviation) of estimates from the maximum likelihood localization of microspheres with a floated width Airy pattern. The results shown are for the same *x*_0_ and *y*_0_ estimates whose averages are analyzed in Fig 5. For each data set, the percentage differences between the x-localization accuracy and the limit of the x-localization accuracy, and between the y-localization accuracy and the limit of the y-localization accuracy, are plotted in green and red if the corresponding absolute differences between the square of the localization accuracy (i.e., the variance of the estimates) and the square of the limit of accuracy are both within 3 and 2 times, respectively, their respective standard errors of the variance for an ideal estimator. The percentages are specified with respect to the limit of accuracy.

The results of fitting with a floated width Airy pattern are presented in Figs 5 and 6, in analogous layout and using the same color coding as in Figs 3 and 4, respectively. On the whole, one can see that floating the width of the Airy pattern allows both standard error-based criteria to be satisfied for essentially all the microsphere sizes considered. There are notable exceptions to the general observation, such as failure of the estimator to satisfy the standard error of the mean criterion for relatively small sizes in the approximate range of 100 nm to 300 nm in the *λ* = 485 nm / 63×, *λ* = 485 nm / 100×, and *λ* = 573 nm / 100× combinations (see plots in Fig 5). These exceptions are reproducible, as they also resulted from the analysis of the second set of statistically identical data sets (see S5 and S6 Figs), as well as from the analysis of the aforementioned data sets with the different microsphere location (see S9 and S10 Figs). Despite the exceptions, which we discuss further in the section *Effect of image pixelation*, it is important to note that the simultaneous estimation of the Airy pattern’s width parameter with the positional coordinates enables the localization of microspheres as large as, or nearly as large as, 1 *μ*m in diameter. Based on the plots of Figs 3 and 4, this is something that cannot be achieved when a fixed width Airy pattern is used for the localization.

An interesting and reproducible phenomenon to take note of is the large percentage difference between the x-localization accuracy and its limit of accuracy for the 50-nm microsphere in the *λ* = 485 nm / 100× combination (see plot in Fig 6). This is unexpected, given the similarity between a 50-nm microsphere’s image and its corresponding point source’s Airy pattern (see, e.g., Fig 1), and their typically comparable limits of the localization accuracy (see., e.g., Fig 2). In this particular case, however, there is a relatively large 13% difference between their limits of the x-localization accuracy, and it is reflected in the significant percentage difference between the obtained accuracy and the limit of accuracy for the microsphere. The unexpected large difference is a function of the particular settings of the *λ* = 485 nm / 100× combination, as it does not show up in the other five combinations considered, or even in the same combination from the data sets with the different microsphere location (see *λ* = 485 nm / 100× plot in S10 Fig), and from data sets with a finer image pixelation (see the section *Effect of image pixelation*). The large difference is also specific to the case where the Airy pattern’s width parameter *α* is estimated along with the positional coordinates. When *α* is fixed, the percentage difference is small as expected (see *λ* = 485 nm / 100× plot in Fig 4).

#### Effect of image pixelation

It can be seen from Fig 3, for the *λ* = 485 nm / 100× combination, that the maximum likelihood estimator that fits a fixed width Airy pattern has difficulty meeting the standard error of the mean criterion for even the smallest of the microsphere sizes considered. Similarly, it can be seen from Fig 5, for the *λ* = 485 nm / 63×, *λ* = 485 nm / 100×, and *λ* = 573 nm / 100× combinations, that the maximum likelihood estimator that fits a floated width Airy pattern fails to meet its standard error of the mean criterion for relatively small microsphere sizes ranging roughly from 100 nm to 300 nm in diameter. Here, we demonstrate that these somewhat unexpected results can at least be partially attributed to the relatively coarse pixelation of the microsphere image data, and can be reversed by using a finer pixelation of the image.

**Fig 7:**
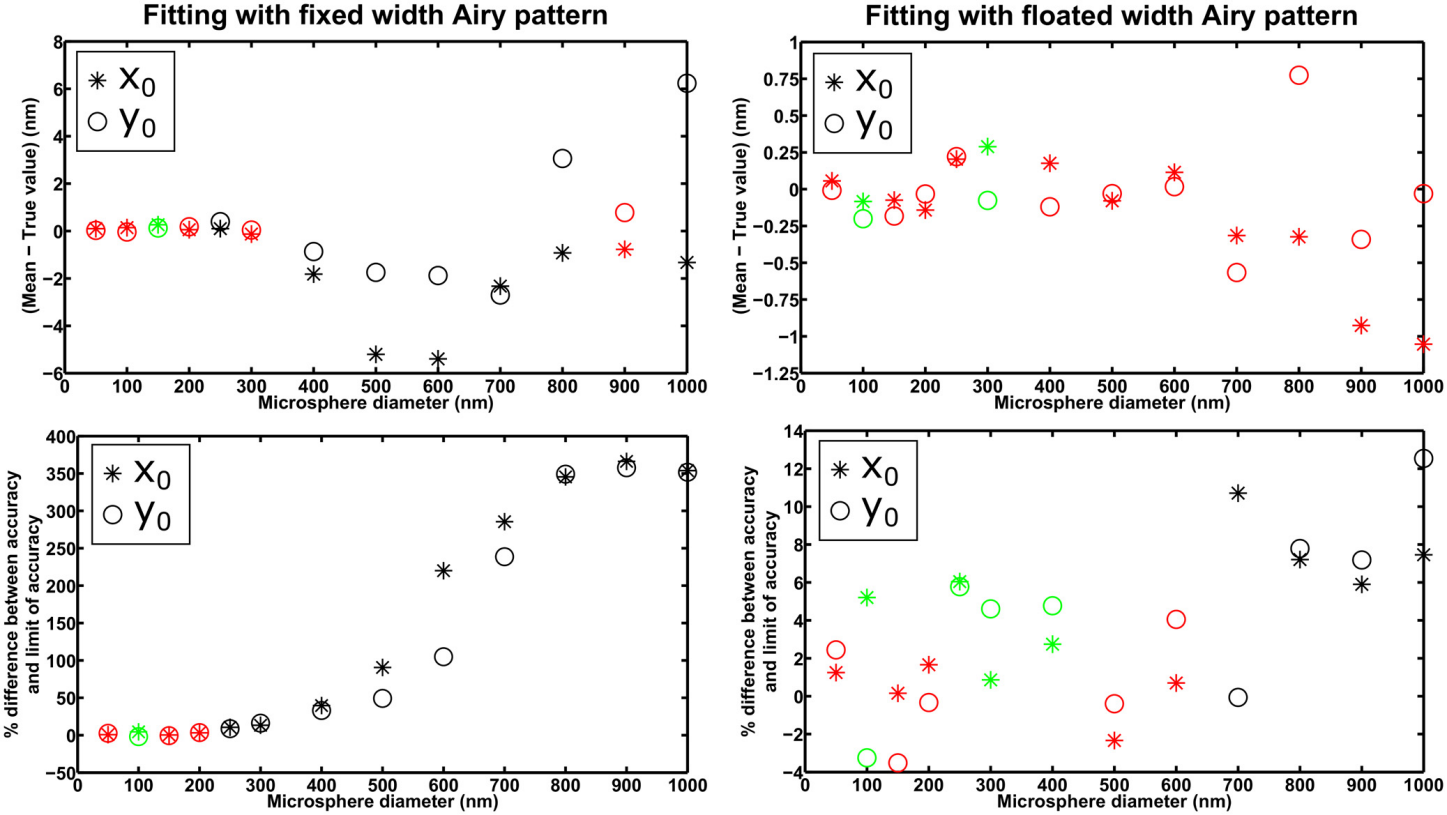
Analysis of the mean and accuracy (i.e., standard deviation) of estimates from the maximum likelihood localization of microspheres with a fixed width (left-hand side plots) and a floated width (right-hand side plots) Airy pattern. Results are shown for 13 data sets, each consisting of 1000 repeat images of a microsphere of a different size, simulated with parameters specified in the section *Simulation parameters* for the *λ* = 485 nm / 100× combination, except the magnification has been changed to *M* = 160 to yield a smaller effective pixel size of 100 nm, the ROI and the per-pixel mean background photon count have accordingly been changed to a 21×21-pixel array and *β*_0_ = 30, respectively, to retain the detection of similar numbers of photons from the microsphere and the background component, and the lateral location of the microsphere has been changed to 10.3 pixels in the *x* direction and 10.1 pixels in the *y* direction within the ROI. For each data set, the difference between the mean of estimates and the true value for each positional coordinate, and the percentage difference between the localization accuracy and the limit of the localization accuracy for each positional coordinate, are color-coded as in Figs 3 through 6.

The 100× and 63× imaging configurations sample the image produced by the microscope with relatively large pixels of effective sizes 160 nm and approximately 205 nm, respectively. By reducing the effective pixel size to 100 nm, and accordingly increasing the ROI to 21×21 pixels and decreasing the mean background photon count to *β*_0_ = 30 photons per pixel to retain approximately the same percentage of captured microsphere photons and roughly the same level of background noise, the maximum likelihood estimators for the above scenarios are able to satisfy their respective standard error of the mean criteria for the small microsphere sizes indicated above. This can be seen in Fig 7, where we show the results for the *λ* = 485 nm / 100× combination, for both fitting with a fixed width, and fitting with a floated width, Airy pattern. These results are again corroborated by those obtained from the analysis of a second set of statistically identical data sets (see S11 Fig). (For similar effects of the 100-nm effective pixel size on the *λ* = 485 nm / 63× and *λ* = 573 nm / 100× combinations, see S12 and S13 Figs)

Note that image pixelation is a factor here because of the mismatch between the data and the fitted Airy pattern. In Table 3, examples are given to demonstrate that by fitting a microsphere image model instead of an Airy pattern to the same problematic data sets indicated above, both standard error-based criteria are satisfied.

**Table 3:** Maximum likelihood microsphere localization with microsphere image model.

Img. Cfg.	*λ* (nm)	*d* (nm)		True value (nm)	Mean of estimates (nm)	Mean − True (nm)	*δ* (nm)	SD (nm)	(SD − *δ*)/*δ* ×100(%)
63×	485	250	*x*_0_	1494.76	1494.70	−0.07*	4.89	5.17	5.76*
			*y*_0_	1453.81	1453.41	−0.40*	5.05	5.21	3.20*
100×	485	100	*x*_0_	1168.00	1167.98	−0.02*	3.17	2.98	−5.90*
			*y*_0_	1136.00	1136.16	0.16*	2.98	3.06	2.97*
100×	485	200	*x*_0_	1168.00	1168.37	0.37^†^	3.61	3.72	3.04*
			*y*_0_	1136.00	1136.41	0.41^†^	3.55	3.67	3.48*
100×	573	200	*x*_0_	1168.00	1168.30	0.30*	4.03	4.03	−0.11*
			*y*_0_	1136.00	1136.04	0.04*	4.13	4.16	0.79*

Results are shown for four of the same simulated microsphere data sets analyzed in Figs 3 through 6. The four data sets differ in terms of imaging configuration, wavelength *λ*, and microsphere diameter *d*, and in each case, only the first 500 images in the data set were considered. Each of the 500 images in a data set was fitted with the microsphere image model used to generate the data set. Only the positional coordinates *x*_0_ and *y*_0_ were estimated. For each data set, the mean and accuracy (i.e., standard deviation SD) of the *x*_0_ and *y*_0_ estimates are shown alongside their respective true values and accuracy limits *δ*. For each coordinate, superscripts † and * for the difference (mean − true) indicate ∣mean − true∣ is between 2 and 3 times, and within 2 times, the standard error of the mean *SE*_*mean*_ for an ideal estimator, respectively. Similarly, superscripts † and * for the % difference between SD and *δ* indicate ∣SD^2^ − *δ* ^2^∣ is between 2 and 3 times, and within 2 times, the standard error of the variance *SE*_*var*_ for an ideal estimator, respectively.

### Experimental data

In the above sections, we used simulated microsphere image data to investigate the suitability of maximum likelihood localization using both fixed width and floated width Airy patterns. Here, we present and discuss the results obtained by carrying out the same types of localization on experimental microsphere image data. A complete view of the acquisition and analysis of the data sets is provided in S4 Text.

Since the true values of the microsphere’s positional coordinates are not known in the case of experimental data, we restrict our discussion to the accuracy of the positional coordinate estimates, for which the corresponding limit of accuracy can be calculated using the estimated values of various parameters. When a fixed width Airy pattern is fitted to the data, the results obtained agree to a large extent with those obtained from the simulated data. Table 4 shows that for a 50-nm microsphere, the estimator is largely able to satisfy the standard error-based criterion. Specifically, for four out of five data sets, the x- and y-localization accuracies are each close enough to its respective limit of accuracy for the percentage difference to be between two and three times († superscript), or even within two times (* superscript), the standard error of the variance. For the remaining data set, the percentage difference for the *x*_0_ coordinate is within two times its corresponding standard error of the variance, but the percentage difference for the *y*_0_ coordinate is greater than three times its corresponding standard error of the variance. In the case of a 100-nm microsphere, the estimator satisfies the criterion for only one out of five data sets, though for each of the other four data sets, the percentage difference for the *x*_0_ coordinate does meet its standard error-based criterion. The performance of the estimator continues to deteriorate with increasing microsphere diameter, to the point where in the case of a 1-*μ*m microsphere, the percentage differences are very large for all five data sets.

**Table 4:** Localization of Fluoresbrite microspheres with a fixed width Airy pattern.

*d* (nm)	Set #	*N*_*photon*_	*β*_0_	2*πn*_*a*_/*λ* (nm^−1^)	*δ*_*x*_, *δ*_*y*_ (nm)	SD_*x*_, SD_*y*_ (nm)	(SD −*δ*) / *δ* × 100 (%)
50	1	653	27.77	0.00933	12.25, 12.25	12.18, 12.77	−0.56*, 4.21*
	2	697	30.57	0.00959	11.36, 11.28	11.56, 11.91	1.80*, 5.59*
	3	1009	29.69	0.00864	9.69, 9.82	9.02, 9.91	−6.95^†^, 0.98*
	4	1014	33.55	0.00911	9.24, 9.05	9.29, 8.83	0.56*, −2.46*
	5	551	33.39	0.00925	14.73, 14.67	14.19, 16.10	−3.65* 9.74
100	1	1248	12.88	0.00910	7.40, 7.40	7.39, 8.44	−0.03*, 14.10
	2	1302	14.37	0.00908	7.26, 7.30	7.71, 8.11	6.27^†^, 10.99
	3	1376	15.25	0.00913	6.94, 6.91	7.14, 7.55	2.94*, 9.27
	4	1208	19.00	0.00933	7.47, 7.71	7.15, 8.73	−4.18*, 13.30
	5	1369	15.90	0.00819	8.04, 8.06	8.56, 8.49	6.48^†^, 5.32*
200 (190)	1	1879	15.56	0.00947	5.63, 5.58	5.83, 6.24	3.57*, 11.82
	2	2203	17.89	0.00951	5.02, 5.04	5.54, 5.93	10.31, 17.78
	3	2684	16.33	0.00917	4.58, 4.55	4.94, 5.18	7.86^†^, 13.88
	4	2255	14.97	0.00852	5.55, 5.58	6.05, 5.86	8.99^†^, 5.00*
	5	2786	14.85	0.00937	4.30, 4.32	4.82, 4.96	12.08, 14.62
300 (320)	1	1939	12.18	0.00832	7.01, 7.07	7.45, 6.92	6.33^†^, −2.14*
	2	2919	12.03	0.00783	5.55, 5.60	6.72, 6.40	21.13, 14.25
	3	2441	12.45	0.00813	6.08, 6.13	9.58, 6.87	57.64, 12.02
	4	2412	11.54	0.00851	5.90, 5.87	7.72, 6.84	30.80, 16.56
	5	1788	11.23	0.00737	8.52, 8.51	10.16, 9.02	19.26, 5.96*
500	1	5644	6.35	0.00736	4.49, 4.49	5.31, 4.88	18.37, 8.73^†^
	2	4131	6.23	0.00729	5.58, 5.59	6.33, 6.46	13.49, 15.60
	3	6345	7.94	0.00717	4.26, 4.26	4.79, 4.92	12.63, 15.73
	4	2261	11.04	0.00601	10.60, 10.61	13.08, 12.76	23.34, 20.28
	5	4686	12.25	0.00592	6.26, 6.26	11.74, 7.47	87.64, 19.29
1000 (908)	1	6569	8.85	0.00470	9.30, 9.30	36.79, 36.22	295.77, 289.58
	2	7105	6.63	0.00580	7.39, 7.39	17.01, 17.19	130.19, 132.68
	3	7761	8.42	0.00435	8.78, 8.77	51.14, 15.94	482.78, 81.68
	4	8459	8.41	0.00411	8.66, 8.66	13.12, 8.13	51.52, −6.10*
	5	8310	7.10	0.00437	8.28, 8.28	27.56, 23.92	232.90, 188.89

Analysis of the accuracy (i.e., the standard deviation SD) of estimates from the maximum likelihood localization of yellow green microspheres with a fixed width Airy pattern. The width parameter of the fitted Airy pattern was fixed to 0.00911 nm^−1^ (see S4 Text). Results are shown for microspheres of six different nominal diameters. For each diameter *d*, each of the five data sets consists of 500 repeat images of a different microsphere. For each data set, the limits of accuracy *δ*_*x*_ and *δ*_*y*_ are calculated with location (*x*_0_, *y*_0_) given by the averages of the *x*_0_ and *y*_0_ estimates (see S1 Table), radius *r* given by half the nominal diameter or average diameter (shown in parentheses if used), and mean microsphere photon count *N*_*photon*_, mean per-pixel background photon count *β*_0_, and parameter $\frac{2\pi n_{a}}{\lambda }$ determined from the sum image (see S4 Text). *N*_*photon*_ and *β*_0_ were also used as fixed values in the localization carried out on each image in the data set. For the % difference between SD and *δ* for each coordinate, superscripts † and * indicate ∣SD^2^ − *δ* ^2^∣ is between 2 and 3 times, and within 2 times, the standard error of the variance *SE*_*var*_ for an ideal estimator, respectively.

The results of Table 4 thus lend further support to the general rule-of-thumb cutoff diameter of 100 nm proposed based on the simulated data, though it would certainly have been more desirable if the estimator satisfied the standard error-based criterion for more of the 100-nm data sets. Moreover, one might have expected better performance in the 200-nm, and even the 300-nm, scenarios, since the experimental data was acquired under conditions similar to those for the *λ* = 573 nm / 63× and *λ* = 663 nm / 63× combinations, which yield good performance in terms of accuracy for microsphere diameters up to at least 200 nm (see plots for these two combinations in Fig 4). Specifically, the experimental setup has the same effective pixel size of approximately 205 nm and an Airy width parameter value of approximately 0.00911 nm^−1^, which is in between the width parameter values for the two combinations (see S2C Fig). The seemingly poorer performance of the estimator in the case of experimental data can perhaps be partially attributed to the fact that the limit of accuracy and the standard error-based criterion used to assess the obtained accuracy are calculated based on a microsphere model that does not perfectly describe the acquired image of a microsphere. Our model, for instance, is simplistic in the sense that it does not account for the effect of the refractive index mismatch between the microsphere sample (which is covered in water), the glass coverslip of the MatTek dish, and the immersion oil. The omission of such effects from the model may play a more significant role as the size of the microsphere increases. This is supported by the fact that the value of the parameter $\frac{2\pi n_{a}}{\lambda }$, estimated using the model from the sum image (see S4 Text), unexpectedly decreases, as opposed to remaining constant, with increasing microsphere size (see Table 4). Other factors potentially contributing to the discrepancy between a given localization accuracy and its limit include the difference between the actual diameter of the particular microsphere and the nominal or average diameter used to compute the limit, and the fact that whereas the imaged microsphere may not have been exactly in focus due to the subjective nature of the manual adjustment of focus, the calculation of the limit assumes it to be in perfect focus. Any inaccuracies in the independent estimation of the microsphere and background photon count levels (see S4 Text) might also have contributed to the discrepancy.

From Table 5, we see that when a floated width Airy pattern is fitted to the same experimental data sets, the results obtained in the case of a 1-*μ*m microsphere corroborate the finding based on our simulated data, demonstrating that the simultaneous estimation of the Airy pattern’s width parameter allows the localization of relatively large microspheres. Though the estimator satisfies the standard error-based criterion for only one out of five data sets, the percentage differences for all five data sets represent marked improvements over those produced by the fitting of a fixed width Airy pattern (see 1-*μ*m results in Table 4). For the smaller diameters ranging from 50 nm to 500 nm, the percentage differences shown in Table 5 are comparable to those shown in Table 4. Based on the plots in Fig 6, which show the estimator to perform well for microsphere sizes up to 1 *μ*m, one might have expected smaller percentage differences for all six sizes considered, and more data sets to meet their standard error-based criterion. However, reasons such as those noted above help to explain the estimator’s poorer performance when it comes to experimental data.

**Table 5:** Localization of Fluoresbrite microspheres with a floated width Airy pattern.

*d*(nm)	Set #	*N*_*photon*_	*β*_0_	2*πn*_*a*_/*λ* (nm^−1^)	*δ*_*x*_, *δ*_*y*_ (nm)	SD_*x*_, SD_*y*_ (nm)	(SD −*δ*) / *δ* × 100 (%)
50	1	653	27.77	0.00933	12.25, 12.25	12.21, 12.87	−0.37*, 5.05*
	2	697	30.57	0.00959	11.36, 11.28	11.52, 11.79	1.46*, 4.48*
	3	1009	29.69	0.00864	9.69, 9.82	9.26, 9.73	−4.45*, −0.90*
	4	1014	33.55	0.00911	9.24, 9.05	9.31, 9.08	0.75*, 0.28*
	5	551	33.39	0.00925	14.73, 14.67	14.35, 16.27	−2.60*, 10.90
100	1	1248	12.88	0.00910	7.40, 7.40	7.50, 8.67	1.41*, 17.19
	2	1302	14.37	0.00908	7.26, 7.31	7.82, 8.13	7.73^†^, 11.20
	3	1376	15.25	0.00913	6.94, 6.91	7.17, 7.66	3.34*, 10.88
	4	1208	19.00	0.00933	7.47, 7.71	7.19, 8.71	−3.69*, 13.05
	5	1369	15.90	0.00819	8.04, 8.06	8.29, 8.80	3.09*, 9.13
200 (190)	1	1879	15.56	0.00947	5.63, 5.58	5.88, 6.34	4.40*, 13.57
	2	2203	17.89	0.00951	5.02, 5.04	5.64, 6.01	12.22, 19.29
	3	2684	16.33	0.00917	4.58, 4.55	5.09, 5.43	11.22, 19.29
	4	2255	14.97	0.00852	5.55, 5.58	5.96, 6.15	7.42^†^, 10.33
	5	2786	14.85	0.00937	4.31, 4.33	4.97, 5.11	15.47, 18.17
300 (320)	1	1939	12.18	0.00832	7.01, 7.07	7.10, 7.25	1.29*, 2.53*
	2	2919	12.03	0.00783	5.55, 5.61	6.58, 6.18	18.56, 10.32
	3	2441	12.45	0.00813	6.08, 6.13	9.86, 7.30	62.08, 19.10
	4	2412	11.54	0.00851	5.90, 5.87	8.09, 6.97	37.05, 18.77
	5	1788	11.23	0.00737	8.52, 8.51	10.57, 9.72	24.07, 14.19
500	1	5644	6.35	0.00736	4.49, 4.49	5.63, 5.34	25.46, 18.87
	2	4131	6.23	0.00729	5.58, 5.59	5.97, 6.50	6.95^†^, 16.30
	3	6345	7.94	0.00717	4.26, 4.25	5.40, 5.12	26.91, 20.27
	4	2261	11.04	0.00601	10.60, 10.61	11.87, 12.11	11.94, 14.17
	5	4686	12.25	0.00592	6.26, 6.26	7.87, 7.67	25.72, 22.50
1000 (908)	1	6569	8.85	0.00470	9.30, 9.30	10.50, 10.18	12.92, 9.54
	2	7105	6.63	0.00580	7.39, 7.39	7.67, 7.41	3.78*, 0.24*
	3	7761	8.42	0.00435	8.77, 8.77	10.66, 9.82	21.54, 11.98
	4	8459	8.41	0.00411	8.66, 8.66	10.79, 10.11	24.59, 16.71
	5	8310	7.10	0.00437	8.28, 8.28	10.11, 9.64	22.14, 16.42

Analysis of the accuracy (i.e., the standard deviation SD) of estimates from the maximum likelihood localization of yellow green microspheres with a floated width Airy pattern. Results are shown for localization carried out on the same data sets from Table 4. All details are as given in Table 4, except that the averages of the *x*_0_ and *y*_0_ estimates used to calculate the limits of accuracy *δ*_*x*_ and *δ*_*y*_ are given in S2 Table.

## Conclusions

To investigate the suitability of localizing a fluorescent microsphere from an image by the fitting of a point spread function, we have analyzed the performance of a maximum likelihood estimator that fits an Airy pattern to the image data. The performance of this estimator has been evaluated based on its ability to recover the true location of a microsphere with the best possible accuracy as determined based on the Cramér-Rao lower bound, which we calculate by modeling the microsphere as a sphere of uniformly distributed fluorescent dye molecules. Specifically, we have carried out estimations on repeat images of a microsphere, and determined suitability of the estimator by assessing the mean and standard deviation of the resulting estimates of the microsphere’s location against criteria based on the standard errors of the mean and the variance for an ideal estimator. From the analysis of data sets simulated for and acquired under standard imaging conditions for single molecule microscopy, we have found that when a fixed width Airy pattern is used to estimate only the microsphere’s positional coordinates, small microspheres of sizes up to 100 nm in diameter can generally be localized with acceptable performance. Furthermore, our results indicate that by allowing the width of the Airy pattern to be estimated along with the positional coordinates, microspheres as large as 1 *μ*m in diameter can also be localized with reasonable performance. Our analysis of simulated data further suggests that under particular settings the estimator may be unsuitable for localizing even the smallest microspheres, and that imaging with smaller effective pixel sizes may help to minimize such occurrences.

## Supporting Information

S1 Fig Limit of the y-localization accuracy as a function of microsphere diameter. Limits are shown for microspheres that emit photons of wavelengths 485 nm, 573 nm, and 663 nm, imaged using the 63× imaging configuration specified in the section *Simulation parameters*. Values of all parameters not explicitly provided here, including the region of interest, the location of the microsphere, and the camera readout noise standard deviation used to compute the limits, are as given in the section *Simulation parameters*. For comparison, the limit of the y-localization accuracy for the point source that is located at the same position, and emits photons of the same wavelength, as the microsphere, is shown at the diameter of 0 nm.
(PDF)

S2 Fig Comparison of images at different wavelengths. Mesh representations of (A) images of 50-nm microspheres, (B) images of 1-*μ*m microspheres, and (C) Airy patterns at wavelengths of 485 nm, 573 nm, and 663 nm are shown overlaid in different colors. The microspheres and point sources are assumed to be imaged using the 63× imaging configuration specified in the section *Simulation parameters*, and the images shown provide the view along the *x*-axis. Values of all relevant parameters not explicitly provided here are as given in the section *Simulation parameters*.
(PDF)

S3 Fig Analysis of the mean of estimates from the maximum likelihood localization of microspheres with a fixed width Airy pattern—second set of data sets statistically identical to the data sets of Figs 3 through 6. Each plot shows the results for 13 data sets, each consisting of 1000 repeat images of a microsphere of a different size, simulated with parameters corresponding to one of six combinations of wavelength and imaging configuration (see the section *Simulation parameters*). Each image in a data set was fitted with an Airy pattern whose positional coordinates *x*_0_ and *y*_0_ were estimated, but whose width parameter *α* was fixed to the value determined by the numerical aperture and wavelength used to generate the data set. For each data set, the differences between the mean of the *x*_0_ estimates and the true value *x*_0_, and between the mean of the *y*_0_ estimates and the true value *y*_0_, are plotted in green and red if both of their magnitudes are within 3 and 2 times, respectively, their respective standard errors of the mean for an ideal estimator.
(PDF)

S4 Fig Analysis of the accuracy (i.e., the standard deviation) of estimates from the maximum likelihood localization of microspheres with a fixed width Airy pattern—second set of data sets statistically identical to the data sets of Figs 3 through 6. The results shown are for the same *x*_0_ and *y*_0_ estimates whose averages are analyzed in S3 Fig. For each data set, the percentage differences between the x-localization accuracy and the limit of the x-localization accuracy, and between the y-localization accuracy and the limit of the y-localization accuracy, are plotted in green and red if the corresponding absolute differences between the square of the localization accuracy (i.e., the variance of the estimates) and the square of the limit of accuracy are both within 3 and 2 times, respectively, their respective standard errors of the variance for an ideal estimator. The percentages are specified with respect to the limit of accuracy.
(PDF)

S5 Fig Analysis of the mean of estimates from the maximum likelihood localization of microspheres with a floated width Airy pattern—second set of data sets statistically identical to the data sets of Figs 3 through 6. The results shown are obtained from localization carried out on the same data sets as in S3 Fig, but with the width parameter of the fitted Airy pattern estimated along with its positional coordinates *x*_0_ and *y*_0_. For each data set, the differences between the mean of the *x*_0_ estimates and the true value *x*_0_, and between the mean of the *y*_0_ estimates and the true value *y*_0_, are plotted in green and red if both of their magnitudes are within 3 and 2 times, respectively, their respective standard errors of the mean for an ideal estimator.
(PDF)

S6 Fig Analysis of the accuracy (i.e., the standard deviation) of estimates from the maximum likelihood localization of microspheres with a floated width Airy pattern—second set of data sets statistically identical to the data sets of Figs 3 through 6. The results shown are for the same *x*_0_ and *y*_0_ estimates whose averages are analyzed in S5 Fig. For each data set, the percentage differences between the x-localization accuracy and the limit of the x-localization accuracy, and between the y-localization accuracy and the limit of the y-localization accuracy, are plotted in green and red if the corresponding absolute differences between the square of the localization accuracy (i.e., the variance of the estimates) and the square of the limit of accuracy are both within 3 and 2 times, respectively, their respective standard errors of the variance for an ideal estimator. The percentages are specified with respect to the limit of accuracy.
(PDF)

S7 Fig Analysis of the mean of estimates from the maximum likelihood localization of microspheres with a fixed width Airy pattern—data sets with a different microsphere location. Each plot shows the results for 13 data sets, each consisting of 1000 repeat images of a microsphere of a different size, simulated with parameters corresponding to one of six combinations of wavelength and imaging configuration specified in the section *Simulation parameters*), except the microsphere location is set to 7.2 pixels in the *x* direction and 7.4 pixels in the *y* direction. Each image in a data set was fitted with an Airy pattern whose positional coordinates *x*_0_ and *y*_0_ were estimated, but whose width parameter *α* was fixed to the value determined by the numerical aperture and wavelength used to generate the data set. For each data set, the differences between the mean of the *x*_0_ estimates and the true value *x*_0_, and between the mean of the *y*_0_ estimates and the true value *y*_0_, are plotted in green and red if both of their magnitudes are within 3 and 2 times, respectively, their respective standard errors of the mean for an ideal estimator.
(PDF)

S8 Fig Analysis of the accuracy (i.e., the standard deviation) of estimates from the maximum likelihood localization of microspheres with a fixed width Airy pattern—data sets with a different microsphere location. The results shown are for the same *x*_0_ and *y*_0_ estimates whose averages are analyzed in S7 Fig. For each data set, the percentage differences between the x-localization accuracy and the limit of the x-localization accuracy, and between the y-localization accuracy and the limit of the y-localization accuracy, are plotted in green and red if the corresponding absolute differences between the square of the localization accuracy (i.e., the variance of the estimates) and the square of the limit of accuracy are both within 3 and 2 times, respectively, their respective standard errors of the variance for an ideal estimator. The percentages are specified with respect to the limit of accuracy.
(PDF)

S9 Fig Analysis of the mean of estimates from the maximum likelihood localization of microspheres with a floated width Airy pattern—data sets with a different microsphere location. The results shown are obtained from localization carried out on the same data sets as in S7 Fig, but with the width parameter of the fitted Airy pattern estimated along with its positional coordinates *x*_0_ and *y*_0_. For each data set, the differences between the mean of the *x*_0_ estimates and the true value *x*_0_, and between the mean of the *y*_0_ estimates and the true value *y*_0_, are plotted in green and red if both of their magnitudes are within 3 and 2 times, respectively, their respective standard errors of the mean for an ideal estimator.
(PDF)

S10 Fig Analysis of the accuracy (i.e., the standard deviation) of estimates from the maximum likelihood localization of microspheres with a floated width Airy pattern—data sets with a different microsphere location. The results shown are for the same *x*_0_ and *y*_0_ estimates whose averages are analyzed in S9 Fig. For each data set, the percentage differences between the x-localization accuracy and the limit of the x-localization accuracy, and between the y-localization accuracy and the limit of the y-localization accuracy, are plotted in green and red if the corresponding absolute differences between the square of the localization accuracy (i.e., the variance of the estimates) and the square of the limit of accuracy are both within 3 and 2 times, respectively, their respective standard errors of the variance for an ideal estimator. The percentages are specified with respect to the limit of accuracy.
(PDF)

S11 Fig Analysis of the mean and accuracy (i.e., standard deviation) of estimates from the maximum likelihood localization of microspheres with a fixed width (left-hand side plots) and a floated width (right-hand side plots) Airy pattern—second set of data sets statistically identical to the data sets of Fig 7. Results are shown for 13 data sets, each consisting of 1000 repeat images of a microsphere of a different size, simulated with parameters specified in the section *Simulation parameters* for the *λ* = 485 nm / 100× combination, except the magnification has been changed to *M* = 160 to yield a smaller effective pixel size of 100 nm, the ROI and the per-pixel mean background photon count have accordingly been changed to a 21×21-pixel array and *β*_0_ = 30, respectively, to retain the detection of similar numbers of photons from the microsphere and the background component, and the lateral location of the microsphere has been changed to 10.3 pixels in the *x* direction and 10.1 pixels in the *y* direction within the ROI. For each data set, the difference between the mean of estimates and the true value for each positional coordinate, and the percentage difference between the localization accuracy and the limit of the localization accuracy for each positional coordinate, are color-coded as in Figs 3 through 6.
(PDF)

S12 Fig Analysis of the mean and accuracy (i.e., standard deviation) of estimates from the maximum likelihood localization of microspheres with a fixed width (left-hand side plots) and a floated width (right-hand side plots) Airy pattern—finer pixelation for the *λ* = 485 nm / 63× combination. Results are shown for 13 data sets, each consisting of 1000 repeat images of a microsphere of a different size, simulated with parameters specified in the section *Simulation parameters* for the *λ* = 485 nm / 63× combination, except the magnification and pixel size have been changed to *M* = 160 and 16 *μ*m × 16 *μ*m to yield a smaller effective pixel size of 100 nm, the ROI and the per-pixel mean background photon count have accordingly been changed to a 21×21-pixel array and *β*_0_ = 30, respectively, to retain the detection of similar numbers of photons from the microsphere and the background component, and the lateral location of the microsphere has been changed to 10.3 pixels in the *x* direction and 10.1 pixels in the *y* direction within the ROI. For each data set, the difference between the mean of estimates and the true value for each positional coordinate, and the percentage difference between the localization accuracy and the limit of the localization accuracy for each positional coordinate, are color-coded as in Figs 3 through 6.
(PDF)

S13 Fig Analysis of the mean and accuracy (i.e., standard deviation) of estimates from the maximum likelihood localization of microspheres with a fixed width (left-hand side plots) and a floated width (right-hand side plots) Airy pattern—finer pixelation for the *λ* = 573 nm / 100× combination. Results are shown for 13 data sets, each consisting of 1000 repeat images of a microsphere of a different size, simulated with parameters specified in the section *Simulation parameters* for the *λ* = 573 nm / 100× combination, except the magnification has been changed to *M* = 160 to yield a smaller effective pixel size of 100 nm, the ROI and the per-pixel mean background photon count have accordingly been changed to a 21×21-pixel array and *β*_0_ = 30, respectively, to retain the detection of similar numbers of photons from the microsphere and the background component, and the lateral location of the microsphere has been changed to 10.3 pixels in the *x* direction and 10.1 pixels in the *y* direction within the ROI. For each data set, the difference between the mean of estimates and the true value for each positional coordinate, and the percentage difference between the localization accuracy and the limit of the localization accuracy for each positional coordinate, are color-coded as in Figs 3 through 6.
(PDF)

S1 Table Averages of the *x*_0_ and *y*_0_ estimates from the localization of Fluoresbrite microspheres with a fixed width Airy pattern. The average values shown pertain to the data sets presented in Table 4.
(PDF)

S2 Table Averages of the *x*_0_ and *y*_0_ estimates from the localization of Fluoresbrite microspheres with a floated width Airy pattern. The average values shown pertain to the data sets presented in Table 5.
(PDF)

S1 Text Larger microspheres are more difficult to localize. (PDF)

S2 Text Longer emission wavelength yields poorer localization accuracy. (PDF)

S3 Text Complete view of simulated data generation and analysis. (PDF)

S4 Text Complete view of experimental data generation and analysis. (PDF)
