# Supplementary material for: Fluorescent Microspheres as Point Sources: A Localization Study
Source: PLoS One. 2015 Jul 28;10(7):e0134112. doi: 10.1371/journal.pone.0134112 (PMC4517909; doi:10.1371/journal.pone.0134112)
Supplement: S1 Fig — Limits are shown for microspheres that emit photons of wavelengths 485 nm, 573 nm, and 663 nm, imaged using the 63× imaging configuration specified in the section Simulation parameters. Values of all parameters not explicitly provided here, including the region of interest, the location of the microsphere, and the camera readout noise standard deviation used to compute the limits, are as given in the section Simulation parameters. For comparison, the limit of the y-localization accuracy for the point source that is located at the same position, and emits photons of the same wavelength, as the microsphere, is shown at the diameter of 0 nm. (PDF) [file pone.0134112.s001.pdf]

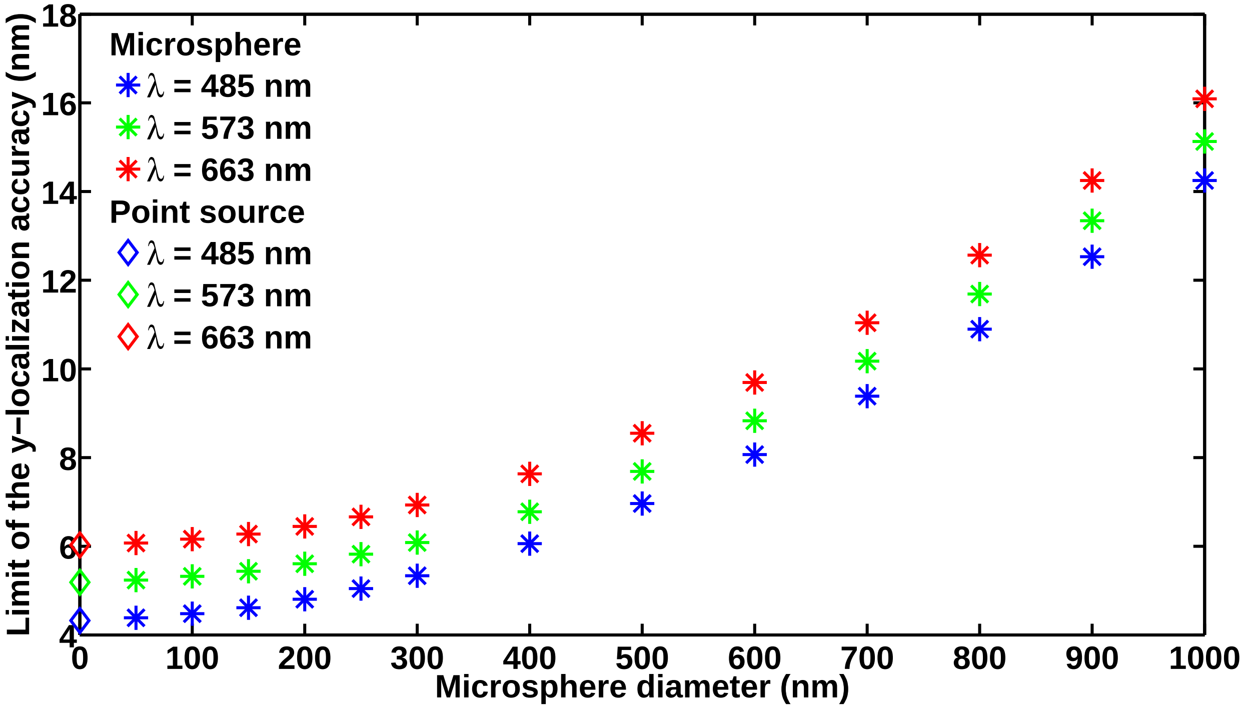

S1 Fig. Limit of the y-localization accuracy as a function of microsphere diameter. Limits are shown for microspheres that emit photons of wavelengths 485 nm, 573 nm, and 663 nm, imaged using the 63× imaging configuration specified in the section *Simulation parameters*. Values of all parameters not explicitly provided here, including the region of interest, the location of the microsphere, and the camera readout noise standard deviation used to compute the limits, are as given in the section *Simulation parameters*. For comparison, the limit of the y-localization accuracy for the point source that is located at the same position, and emits photons of the same wavelength, as the microsphere, is shown at the diameter of 0 nm.
