# Supplementary material for: Fluorescent Microspheres as Point Sources: A Localization Study
Source: PLoS One. 2015 Jul 28;10(7):e0134112. doi: 10.1371/journal.pone.0134112 (PMC4517909; doi:10.1371/journal.pone.0134112)
Supplement: S2 Fig — Mesh representations of (A) images of 50-nm microspheres, (B) images of 1-μm microspheres, and (C) Airy patterns at wavelengths of 485 nm, 573 nm, and 663 nm are shown overlaid in different colors. The microspheres and point sources are assumed to be imaged using the 63× imaging configuration specified in the section Simulation parameters, and the images shown provide the view along the x-axis. Values of all relevant parameters not explicitly provided here are as given in the section Simulation parameters. (PDF) [file pone.0134112.s002.pdf]

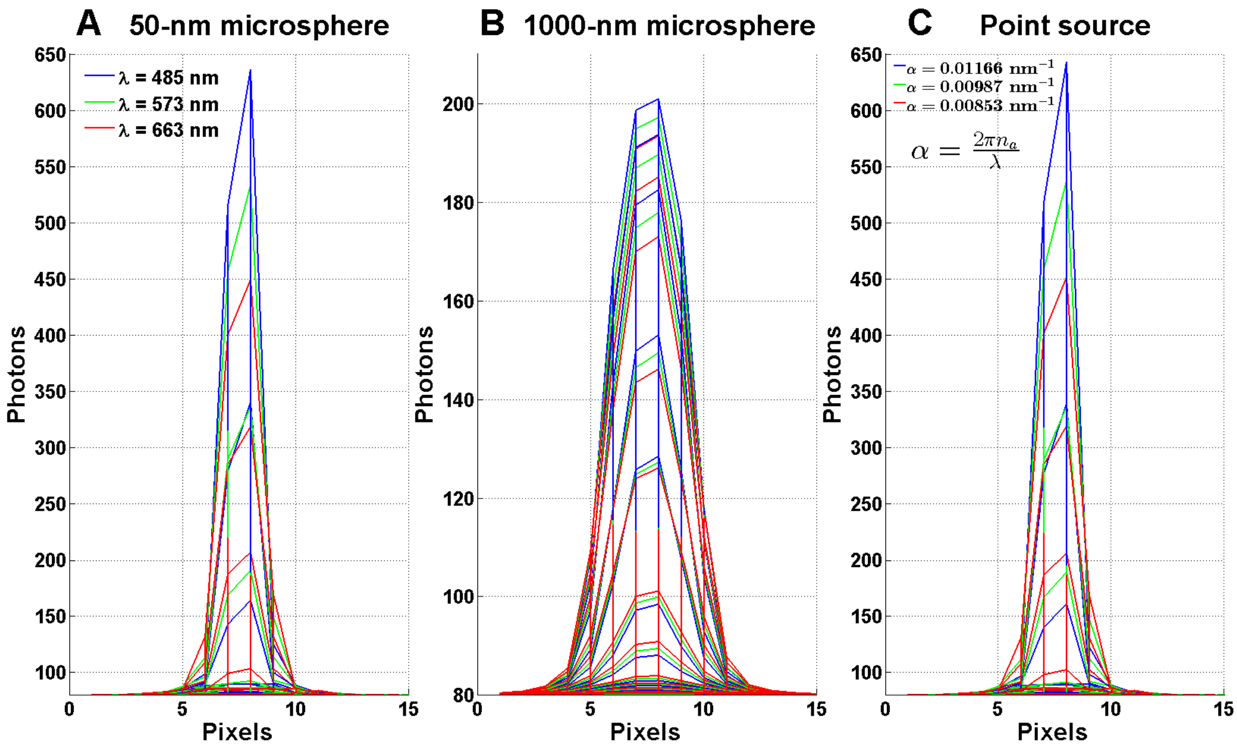

S2 Fig. Comparison of images at different wavelengths. Mesh representations of (A) images of 50-nm microspheres, (B) images of 1-μm microspheres, and (C) Airy patterns at wavelengths of 485 nm, 573 nm, and 663 nm are shown overlaid in different colors. The microspheres and point sources are assumed to be imaged using the 63× imaging configuration specified in the section *Simulation parameters*, and the images shown provide the view along the x-axis. Values of all relevant parameters not explicitly provided here are as given in the section *Simulation parameters*.
