# Supplementary material for: Fluorescent Microspheres as Point Sources: A Localization Study
Source: PLoS One. 2015 Jul 28;10(7):e0134112. doi: 10.1371/journal.pone.0134112 (PMC4517909; doi:10.1371/journal.pone.0134112)
Supplement: S3 Fig — Each plot shows the results for 13 data sets, each consisting of 1000 repeat images of a microsphere of a different size, simulated with parameters corresponding to one of six combinations of wavelength and imaging configuration (see the section Simulation parameters). Each image in a data set was fitted with an Airy pattern whose positional coordinates x 0 and y 0 were estimated, but whose width parameter α was fixed to the value determined by the numerical aperture and wavelength used to generate the data set. For each data set, the differences between the mean of the x 0 estimates and the true value x 0, and between the mean of the y 0 estimates and the true value y 0, are plotted in green and red if both of their magnitudes are within 3 and 2 times, respectively, their respective standard errors of the mean for an ideal estimator. (PDF) [file pone.0134112.s003.pdf]

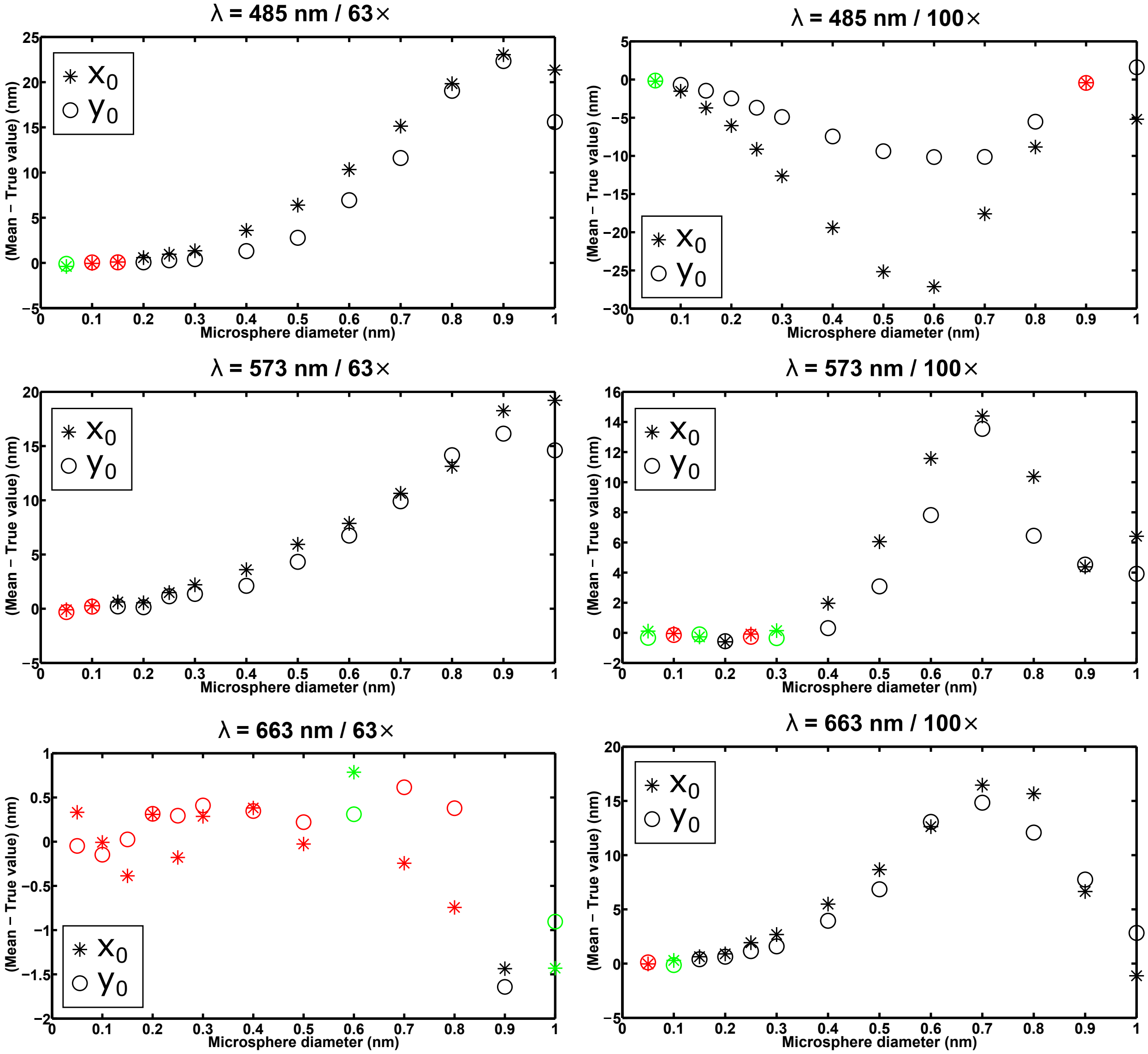

S3 Fig. Analysis of the mean of estimates from the maximum likelihood localization of microspheres with a fixed width Airy pattern - second set of data sets statistically identical to the data sets of Figs. 3 through 6. Each plot shows the results for 13 data sets, each consisting of 1000 repeat images of a microsphere of a different size, simulated with parameters corresponding to one of six combinations of wavelength and imaging configuration (see the section *Simulation parameters*). Each image in a data set was fitted with an Airy pattern whose positional coordinates x_0 and y_0 were estimated, but whose width parameter α was fixed to the value determined by the numerical aperture and wavelength used to generate the data set. For each data set, the differences between the mean of the x_0 estimates and the true value x_0, and between the mean of the y_0 estimates and the true value y_0, are plotted in green and red if both of their magnitudes are within 3 and 2 times, respectively, their respective standard errors of the mean for an ideal estimator.
