# Supplementary material for: Fluorescent Microspheres as Point Sources: A Localization Study
Source: PLoS One. 2015 Jul 28;10(7):e0134112. doi: 10.1371/journal.pone.0134112 (PMC4517909; doi:10.1371/journal.pone.0134112)
Supplement: S5 Fig — The results shown are obtained from localization carried out on the same data sets as in S3 Fig, but with the width parameter of the fitted Airy pattern estimated along with its positional coordinates x 0 and y 0. For each data set, the differences between the mean of the x 0 estimates and the true value x 0, and between the mean of the y 0 estimates and the true value y 0, are plotted in green and red if both of their magnitudes are within 3 and 2 times, respectively, their respective standard errors of the mean for an ideal estimator. (PDF) [file pone.0134112.s005.pdf]

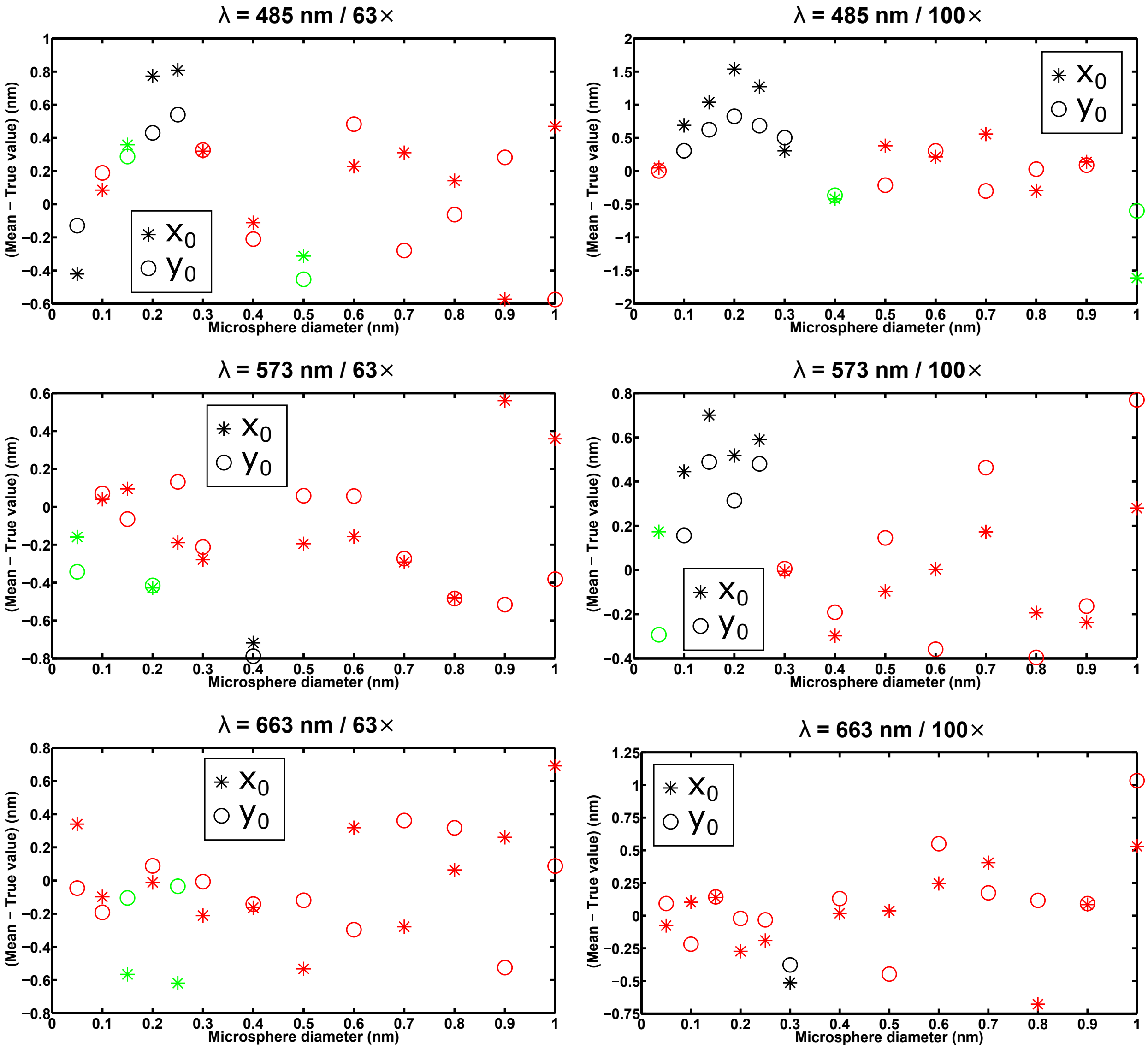

S5 Fig. Analysis of the mean of estimates from the maximum likelihood localization of microspheres with a floated width Airy pattern - second set of data sets statistically identical to the data sets of Figs. 3 through 6. The results shown are obtained from localization carried out on the same data sets as in S3 Fig, but with the width parameter of the fitted Airy pattern estimated along with its positional coordinates x_0 and y_0. For each data set, the differences between the mean of the x_0 estimates and the true value x_0, and between the mean of the y_0 estimates and the true value y_0, are plotted in green and red if both of their magnitudes are within 3 and 2 times, respectively, their respective standard errors of the mean for an ideal estimator.
