# Supplementary material for: Fluorescent Microspheres as Point Sources: A Localization Study
Source: PLoS One. 2015 Jul 28;10(7):e0134112. doi: 10.1371/journal.pone.0134112 (PMC4517909; doi:10.1371/journal.pone.0134112)
Supplement: S7 Fig — Each plot shows the results for 13 data sets, each consisting of 1000 repeat images of a microsphere of a different size, simulated with parameters corresponding to one of six combinations of wavelength and imaging configuration specified in the section Simulation parameters), except the microsphere location is set to 7.2 pixels in the x direction and 7.4 pixels in the y direction. Each image in a data set was fitted with an Airy pattern whose positional coordinates x 0 and y 0 were estimated, but whose width parameter α was fixed to the value determined by the numerical aperture and wavelength used to generate the data set. For each data set, the differences between the mean of the x 0 estimates and the true value x 0, and between the mean of the y 0 estimates and the true value y 0, are plotted in green and red if both of their magnitudes are within 3 and 2 times, respectively, their respective standard errors of the mean for an ideal estimator. (PDF) [file pone.0134112.s007.pdf]

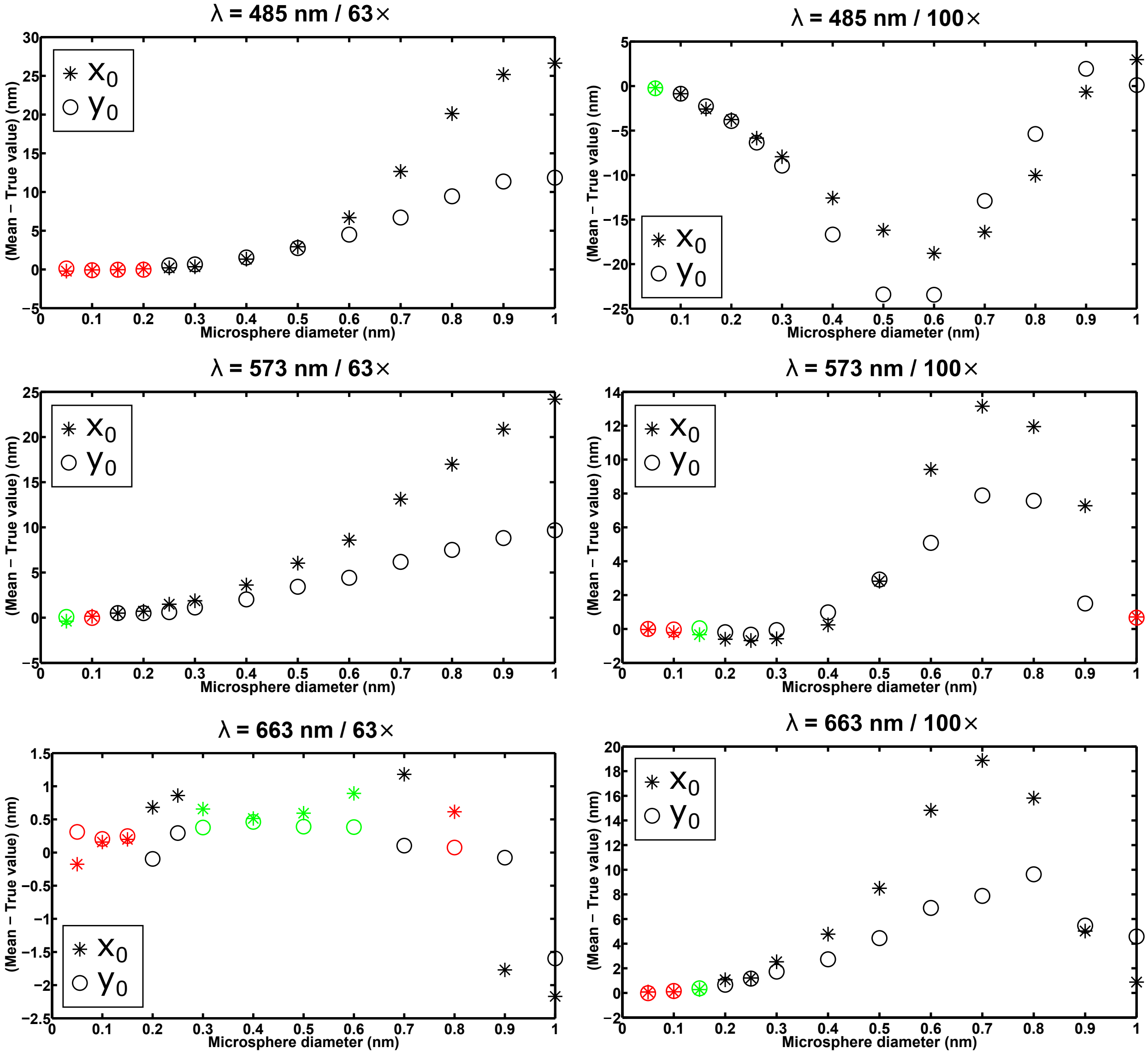

S7 Fig. Analysis of the mean of estimates from the maximum likelihood localization of microspheres with a fixed width Airy pattern - data sets with a different microsphere location. Each plot shows the results for 13 data sets, each consisting of 1000 repeat images of a microsphere of a different size, simulated with parameters corresponding to one of six combinations of wavelength and imaging configuration specified in the section *Simulation parameters*), except the microsphere location is set to 7.2 pixels in the x direction and 7.4 pixels in the y direction. Each image in a data set was fitted with an Airy pattern whose positional coordinates x_0 and y_0 were estimated, but whose width parameter α was fixed to the value determined by the numerical aperture and wavelength used to generate the data set. For each data set, the differences between the mean of the x_0 estimates and the true value x_0, and between the mean of the y_0 estimates and the true value y_0, are plotted in green and red if both of their magnitudes are within 3 and 2 times, respectively, their respective standard errors of the mean for an ideal estimator.
