# Supplementary material for: Fluorescent Microspheres as Point Sources: A Localization Study
Source: PLoS One. 2015 Jul 28;10(7):e0134112. doi: 10.1371/journal.pone.0134112 (PMC4517909; doi:10.1371/journal.pone.0134112)
Supplement: S10 Fig — The results shown are for the same x 0 and y 0 estimates whose averages are analyzed in S9 Fig. For each data set, the percentage differences between the x-localization accuracy and the limit of the x-localization accuracy, and between the y-localization accuracy and the limit of the y-localization accuracy, are plotted in green and red if the corresponding absolute differences between the square of the localization accuracy (i.e., the variance of the estimates) and the square of the limit of accuracy are both within 3 and 2 times, respectively, their respective standard errors of the variance for an ideal estimator. The percentages are specified with respect to the limit of accuracy. (PDF) [file pone.0134112.s010.pdf]

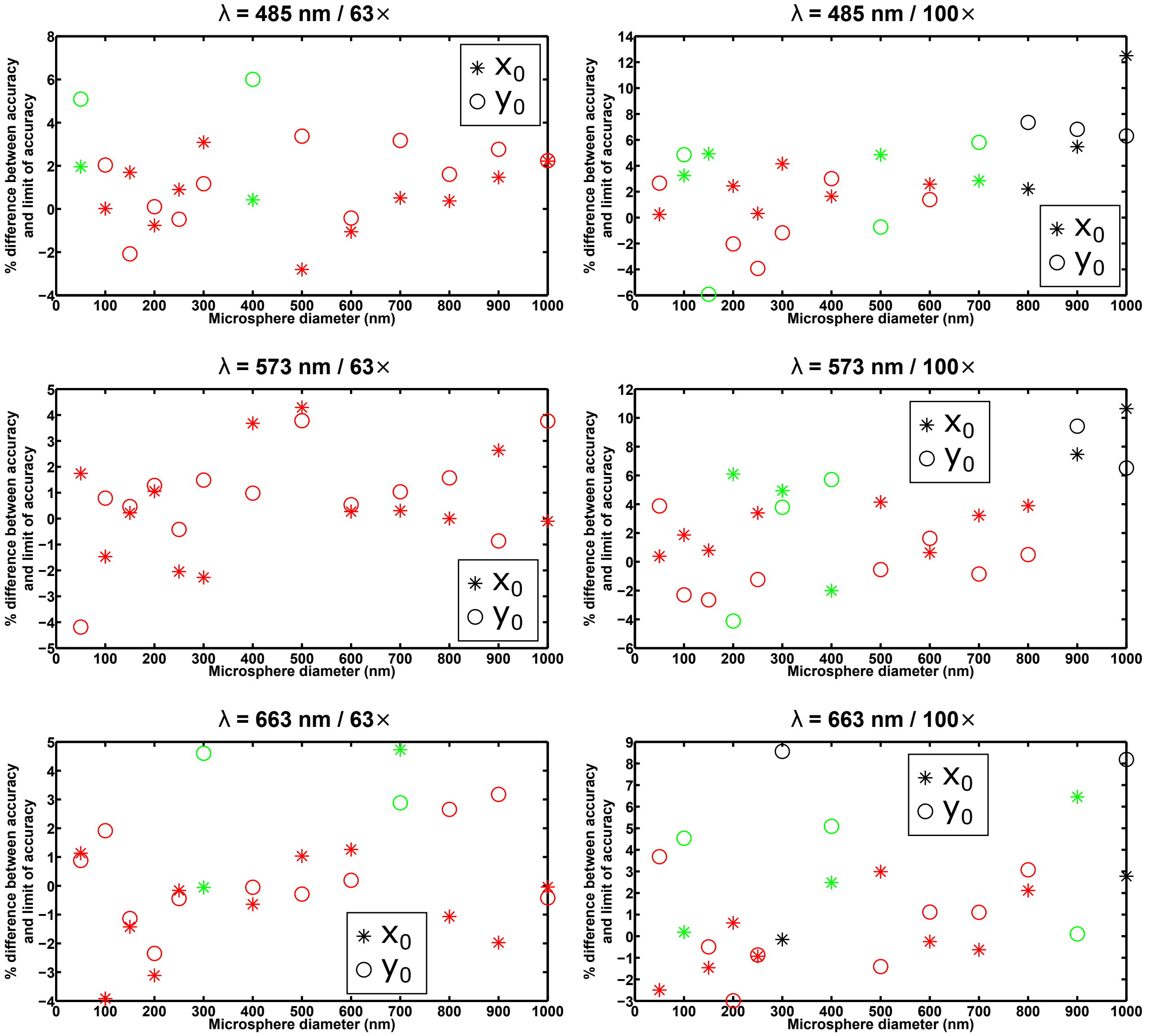

S10 Fig. Analysis of the accuracy (i.e., the standard deviation) of estimates from the maximum likelihood localization of microspheres with a floated width Airy pattern - data sets with a different microsphere location. The results shown are for the same x_0 and y_0 estimates whose averages are analyzed in S9 Fig. For each data set, the percentage differences between the x-localization accuracy and the limit of the x-localization accuracy, and between the y-localization accuracy and the limit of the y-localization accuracy, are plotted in green and red if the corresponding absolute differences between the square of the localization accuracy (i.e., the variance of the estimates) and the square of the limit of accuracy are both within 3 and 2 times, respectively, their respective standard errors of the variance for an ideal estimator. The percentages are specified with respect to the limit of accuracy.
