# Supplementary material for: Fluorescent Microspheres as Point Sources: A Localization Study
Source: PLoS One. 2015 Jul 28;10(7):e0134112. doi: 10.1371/journal.pone.0134112 (PMC4517909; doi:10.1371/journal.pone.0134112)
Supplement: S13 Fig — Results are shown for 13 data sets, each consisting of 1000 repeat images of a microsphere of a different size, simulated with parameters specified in the section Simulation parameters for the λ = 573 nm / 100× combination, except the magnification has been changed to M = 160 to yield a smaller effective pixel size of 100 nm, the ROI and the per-pixel mean background photon count have accordingly been changed to a 21×21-pixel array and β 0 = 30, respectively, to retain the detection of similar numbers of photons from the microsphere and the background component, and the lateral location of the microsphere has been changed to 10.3 pixels in the x direction and 10.1 pixels in the y direction within the ROI. For each data set, the difference between the mean of estimates and the true value for each positional coordinate, and the percentage difference between the localization accuracy and the limit of the localization accuracy for each positional coordinate, are color-coded as in Figs 3 through 6. (PDF) [file pone.0134112.s013.pdf]

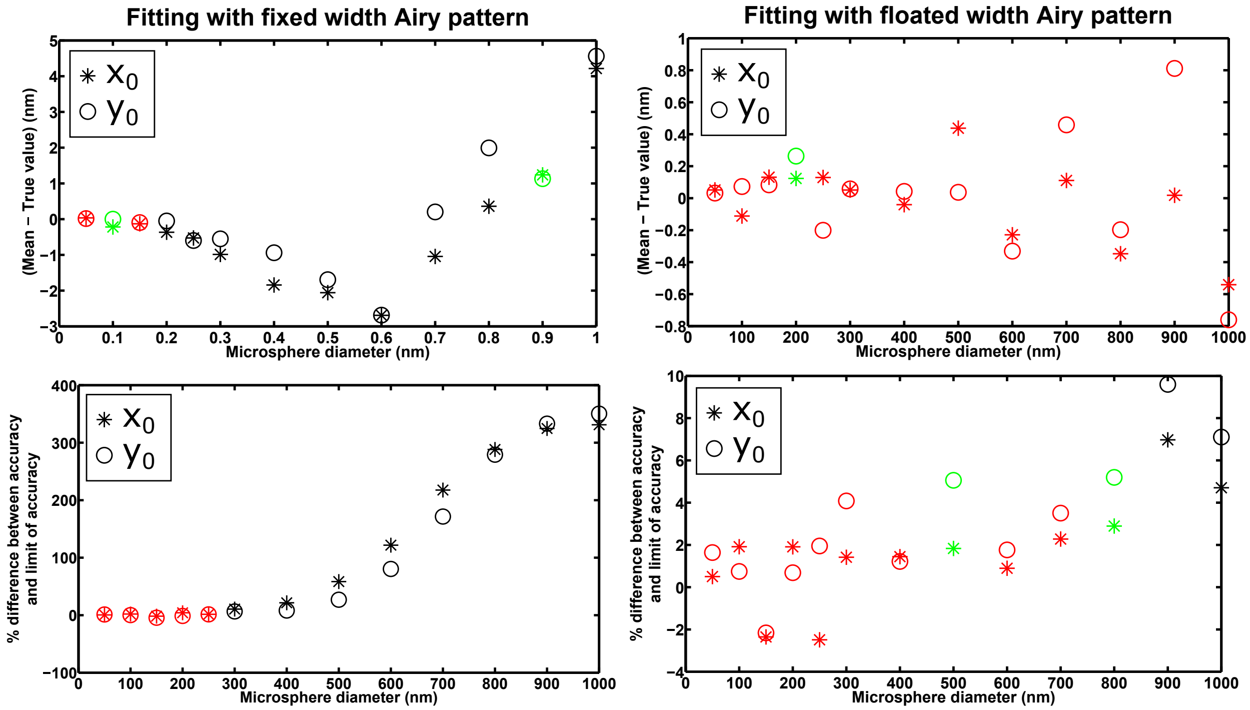

S13 Fig. Analysis of the mean and accuracy (i.e., standard deviation) of estimates from the maximum likelihood localization of microspheres with a fixed width (left-hand side plots) and a floated width (right-hand side plots) Airy pattern - finer pixelation for the λ = 573 nm / 100× combination. Results are shown for 13 data sets, each consisting of 1000 repeat images of a microsphere of a different size, simulated with parameters specified in the section *Simulation parameters* for the λ = 573 nm / 100× combination, except the magnification has been changed to $M = 160$ to yield a smaller effective pixel size of 100 nm, the ROI and the per-pixel mean background photon count have accordingly been changed to a 21×21-pixel array and $\beta_0 = 30$, respectively, to retain the detection of similar numbers of photons from the microsphere and the background component, and the lateral location of the microsphere has been changed to 10.3 pixels in the x direction and 10.1 pixels in the y direction within the ROI. For each data set, the difference between the mean of estimates and the true value for each positional coordinate, and the percentage difference between the localization accuracy and the limit of the localization accuracy for each positional coordinate, are color-coded as in Figs. 3 through 6.
