# Supplementary material for: Fluorescent Microspheres as Point Sources: A Localization Study
Source: PLoS One. 2015 Jul 28;10(7):e0134112. doi: 10.1371/journal.pone.0134112 (PMC4517909; doi:10.1371/journal.pone.0134112)
Supplement: S1 Table — The average values shown pertain to the data sets presented in Table 4. (PDF) [file pone.0134112.s014.pdf]

S1 Table. Averages of the x_0 and y_0 estimates from the localization of Fluoresbrite microspheres with a fixed width Airy pattern.

Microsphere diameter (nm)	Data Set #	Mean of x_0 estimates (nm)	Mean of y_0 estimates (nm)
50	1	1537.69	1533.70
	2	1562.22	1528.57
	3	1552.94	1466.64
	4	1599.32	1559.95
	5	1577.28	1569.80
100	1	1536.66	1537.62
	2	1510.53	1578.71
	3	1565.68	1550.60
	4	1523.35	1608.88
	5	1612.13	1498.62
200 (190)	1	1576.14	1523.46
	2	1569.37	1577.35
	3	1611.50	1536.93
	4	1591.09	1521.24
	5	1544.32	1577.02
300 (320)	1	1639.24	1530.42
	2	1456.59	1522.76
	3	1459.01	1506.02
	4	1532.00	1585.90
	5	1521.72	1560.48
500	1	1627.25	1612.68
	2	1529.42	1632.22
	3	1464.13	1601.27
	4	1533.33	1480.54
	5	1536.31	1476.85
1000 (908)	1	1513.19	1640.41
	2	1607.35	1606.11
	3	1677.91	1556.38
	4	1562.32	1536.24
	5	1568.61	1499.40

The average values shown pertain to the data sets presented in Table 4.
