# Supplementary material for: Fluorescent Microspheres as Point Sources: A Localization Study
Source: PLoS One. 2015 Jul 28;10(7):e0134112. doi: 10.1371/journal.pone.0134112 (PMC4517909; doi:10.1371/journal.pone.0134112)
Supplement: S2 Table — The average values shown pertain to the data sets presented in Table 5. (PDF) [file pone.0134112.s015.pdf]

S2 Table. Averages of the x_0 and y_0 estimates from the localization of Fluoresbrite microspheres with a floated width Airy pattern.

Microsphere diameter (nm)	Data Set #	Mean of x_0 estimates (nm)	Mean of y_0 estimates (nm)
50	1	1537.54	1533.93
	2	1562.21	1528.90
	3	1553.07	1466.18
	4	1599.58	1560.45
	5	1577.10	1569.31
100	1	1536.71	1537.71
	2	1510.39	1579.69
	3	1565.79	1550.67
	4	1523.44	1608.54
	5	1613.26	1497.54
200 (190)	1	1576.65	1523.36
	2	1569.94	1578.11
	3	1611.98	1537.23
	4	1593.33	1522.03
	5	1544.86	1578.83
300 (320)	1	1639.36	1531.62
	2	1456.66	1526.02
	3	1460.50	1509.30
	4	1533.11	1588.27
	5	1519.92	1565.25
500	1	1624.69	1612.24
	2	1528.73	1636.19
	3	1466.82	1599.17
	4	1536.91	1483.24
	5	1540.11	1491.10
1000 (908)	1	1516.16	1635.19
	2	1585.78	1572.54
	3	1654.43	1588.06
	4	1585.59	1570.04
	5	1586.63	1491.37

The average values shown pertain to the data sets presented in Table 5.
