# Supplementary material for: Fluorescent Microspheres as Point Sources: A Localization Study
Source: PLoS One. 2015 Jul 28;10(7):e0134112. doi: 10.1371/journal.pone.0134112 (PMC4517909; doi:10.1371/journal.pone.0134112)
Supplement: S1 Text — (PDF) [file pone.0134112.s016.pdf]

S1 Text

Larger microspheres are more difficult to localize

Fig. 2 shows that for a given color, the limit of the localization accuracy deteriorates (i.e., increases in value) with increasing microsphere diameter, assuming the average number of microsphere photons detected over the detector plane is the same regardless of the microsphere's size. This is expected because a larger diameter yields a broader image profile (see, e.g., Fig. 1), and a broader image profile makes it more difficult to pinpoint the position of the underlying object. The latter assertion is supported, for example, by the simple expressions given in [13, 16] for the limit of the accuracy for localizing an in-focus point source imaged under ideal conditions. These expressions clearly show that the best possible accuracy for localizing the point source worsens when the width of its image profile, modeled as an Airy pattern or a 2-dimensional Gaussian function, is increased. Intuitively, one can also see that a higher localization uncertainty should be expected with a broader image profile because the positional coordinates of the underlying object lie within, and therefore must be extracted from, a flatter peak of some 2-dimensional surface. Note, however, that while larger microspheres may be more difficult to localize than smaller microspheres given the same average photon count, in practice more photons can typically be detected from larger microspheres under the same experimental conditions to significantly improve the localization accuracy (see, e.g., the experimental results reported in Tables 4 and 5). The fact that the estimation accuracy can be improved by detecting more photons from the object of interest is a well-known result [15, 16].
