# Supplementary material for: Fluorescent Microspheres as Point Sources: A Localization Study
Source: PLoS One. 2015 Jul 28;10(7):e0134112. doi: 10.1371/journal.pone.0134112 (PMC4517909; doi:10.1371/journal.pone.0134112)
Supplement: S2 Text — (PDF) [file pone.0134112.s017.pdf]

S2 Text

Longer emission wavelength yields poorer localization accuracy

For a given microsphere diameter, it can be seen in Fig. 2 that the longer the wavelength of the photons detected from the microsphere, the worse the limit of the localization accuracy. This can be attributed to the fact that increasing the wavelength broadens the image profile of the microsphere, thereby making the microsphere's position more difficult to determine (see S1 Text). The broadening of the image profile with increasing wavelength is illustrated in S2 Fig. A and S2 Fig. B for a 50-nm and a 1-μm microsphere, respectively. In each case, the broadening of the profile can perhaps be better appreciated by observing the decrease in the height of the image profile as the wavelength is increased.

Fig. 2 further demonstrates that for smaller microspheres, the limit of the localization accuracy depends on the wavelength in a more substantial way. The x_0 coordinate of a 200-nm microsphere, for example, can be estimated with a best possible accuracy of 4.64 nm when the microsphere emits photons of wavelength 485 nm, but with a best possible accuracy of only 6.45 nm when photons of the longer wavelength 663 nm are emitted. The latter accuracy is worse than the former by 39%. Conversely, for larger microspheres, the limit of accuracy depends on the wavelength to a lesser extent. For the largest diameter of 1 μm considered here, for example, the best possible accuracy of 16.09 nm at the 663-nm wavelength is worse than the best possible accuracy of 14.24 nm at the 485-nm wavelength by a considerably smaller 13%.

The greater effect of wavelength at the smaller sizes is a result of the more significant change in the width (readily seen as a more significant change in the height) of the image profile when the wavelength is changed. This is evident when comparing the relatively big differences in the heights of the three image profiles for the 50-nm microsphere in S2 Fig. A with the relatively small differences in the heights of the three profiles for the 1-μm microsphere in S2 Fig. B. The more pronounced effect for a small microsphere is expected based on its image's similarity to an Airy pattern, the width of which is determined by the wavelength via its width parameter. S2 Fig. C, for example, shows the value of an Airy pattern's width parameter to decrease with increasing wavelength, resulting in a broadening of the pattern. Note how closely the 50-nm microsphere's profiles in S2 Fig. A resemble the Airy patterns in S2 Fig. C.

The relatively small effect of wavelength on the accuracy for localizing larger microspheres is due to the width of the image of a large microsphere being more a function of the microsphere's physical dimensions than a function of the wavelength of the microsphere's photons. This can be seen in S2 Fig. B, which again illustrates that changing the wavelength does relatively little in terms of altering the width of the image of a 1-μm microsphere. A more intuitive explanation is simply that the color of a relatively large object has little effect on the shape of the object's image, and hence little effect on how well the position of the object can be estimated.
