# Supplementary material for: Fluorescent Microspheres as Point Sources: A Localization Study
Source: PLoS One. 2015 Jul 28;10(7):e0134112. doi: 10.1371/journal.pone.0134112 (PMC4517909; doi:10.1371/journal.pone.0134112)
Supplement: S3 Text — (PDF) [file pone.0134112.s018.pdf]

S3 Text

Complete view of simulated data generation and analysis

Simulated microsphere and point source data sets were generated using the procedure of the section *Image simulation*, with settings as specified for the six combinations of wavelength and imaging configuration detailed in the section *Simulation parameters*. Each image in a given data set was subjected to maximum likelihood localization that fits an Airy pattern to the image, the details of which are given in the section *Maximum likelihood localization*. After discarding any pairs of x_0 and y_0 estimates that place the microsphere or point source outside the ROI used for the localization, the mean of the x_0 estimates and the mean of the y_0 estimates were calculated, and respectively compared to the true values x_0 and y_0. Likewise, the x-localization accuracy and the y-localization accuracy for the data set were calculated as the standard deviations of the x_0 estimates and the y_0 estimates, and respectively compared to the limits of the x- and y-localization accuracy, computed as described in the sections *Localization accuracy and its limit* and *The Airy pattern* for microsphere data and point source data, respectively.

Two types of localization were carried out on each data set. In one case, the width parameter of the Airy pattern was not estimated during the maximum likelihood localization, and was instead fixed to its theoretical value of $\frac{2\pi n_a}{\lambda}$, with n_a and λ as determined by the assumed imaging settings. In the other case, the width parameter was estimated along with the positional coordinates of the microsphere or point source, such that the maximum likelihood estimator was able to narrow or broaden the Airy pattern.

In both cases, the initial guesses for x_0 and y_0 were randomly generated, for each image in the data set, to be within ±15% of x_0 and y_0, respectively. This translates to initial guesses that are within approximately one pixel of the true value in both the x and y directions. In the case where the Airy width parameter was estimated, the initial guess for the width parameter was likewise randomly generated, for each image in the data set, to be within ±15% of the true (i.e., theoretical) value in the analysis of point source data sets. In the analysis of microsphere data sets, however, the initial guess for the Airy width parameter was the same for every image in the data set, and was determined by visually matching an Airy pattern to the model image of the microsphere.
