# Supplementary material for: Fluorescent Microspheres as Point Sources: A Localization Study
Source: PLoS One. 2015 Jul 28;10(7):e0134112. doi: 10.1371/journal.pone.0134112 (PMC4517909; doi:10.1371/journal.pone.0134112)
Supplement: S4 Text — (PDF) [file pone.0134112.s019.pdf]

S4 Text

Complete view of experimental data generation and analysis

Each experimental data set consisted of repeat images of an in-focus microsphere, acquired as described in the section *Experimental image acquisition*. Prior to analysis, each image's pixel values were converted to units of photons by subtracting the camera offset and then multiplying by the gain conversion factor.

As in the case of the simulated data, each image in a given experimental data set was subjected to the maximum likelihood fitting of an Airy pattern as described in the section *Maximum likelihood localization*, and the x- and y-localization accuracies computed from the x_0 and y_0 estimates (after elimination of any pairs of x_0 and y_0 estimates that place the microsphere outside the ROI used for the localization) were respectively compared to the limits of the x- and y-localization accuracy (see the section *Localization accuracy and its limit*). More precisely, the localization was performed on a 15×15-pixel ROI on which the image of the microsphere was approximately centered, and the limits of accuracy, too, were calculated based on the ROI. Note, however, that the localization accuracies and their corresponding limits were computed based on estimates that were first corrected for drift of the microsphere sample with respect to the CCD camera. Drift-corrected x_0 estimates were obtained by fitting a cubic polynomial to the x_0 estimates to generate a drift curve, and then subtracting that curve from the x_0 estimates. Drift-corrected y_0 estimates were obtained independently in analogous fashion.

Also as in the case of the simulated data, each experimental data set was localized in two different ways - with and without the estimation of the Airy pattern's width parameter along with the positional coordinates of the microsphere. In the latter case, the width parameter of the Airy pattern was fixed to 0.00911 nm^{-1}, the average value of the width parameters estimated in the former case from the five 50-nm microsphere data sets. This average value makes a reasonable choice because of the high degree of similarity between the image of a 50-nm microsphere and the image of its corresponding point source.

For every image in a data set, the initial guesses for x_0 and y_0 were set to be the coordinates of the center of the ROI. In the case where the Airy width parameter was estimated, with the exception of the 1-μm microsphere data sets, the initial guess for the width parameter was set to the theoretical value $\frac{2\pi n_a}{\lambda}$, with $n_a = 1.4$ and $\lambda = 485$ nm, based on the objective lens and emission filter used for the image acquisition (see the section *Experimental image acquisition*). For the 1-μm microsphere data sets, the initial guess was set to the much broader width of 0.003 nm^{-1}, as we found the estimator to have trouble broadening the Airy pattern to match the large width of the 1-μm microsphere when the theoretical value was used as the initial guess.

Unlike for the simulated data sets where values of parameters that are not estimated, but are required for the maximum likelihood localization, are known, for the experimental data sets the values of these parameters need to be separately determined. Values for the image pixel size, the objective magnification M, the refractive index n of the lens immersion medium, and

the mean η_0 and standard deviation σ_0 of the CCD camera's readout noise all depend solely on the acquisition setup and environment, and are given in the section *Experimental image acquisition*. Values for the mean photon count N_{photon} detected from a microsphere, and the mean photon count β_0 detected per pixel from the background component, however, depend on the particular microsphere sample in addition to the acquisition setup and environment, and were determined for each microsphere data set individually as follows.

For each experimental data set, we first verified that a constant signal level and a constant background level could be reasonably assumed over the course of time during which the data set was acquired. To do so, an ROI (not the relatively small 15×15-pixel ROI subsequently used for the maximum likelihood localization) was selected around the microsphere. This ROI was chosen to be as large as possible (without coming too close to a neighboring microsphere) in order to get a good approximation of the background level based on the values of the peripheral pixels of the ROI. Specifically, for each image in the data set, the background level was approximated as the average of the values of the pixels on the outermost rows and columns of the ROI. The stability of the rate at which background photons were detected during the data acquisition was then visually verified as a relatively even spread of points about their mean in a scatter plot of the background levels determined for the set of images. By the assumption of a spatially uniform background (justified by the relatively flat periphery of the ROI), for each image in the data set an approximation of the number of microsphere photons detected within the ROI was obtained by multiplying the background level for the image by the number of pixels comprising the ROI, and subtracting the result from the sum of the values of all pixels comprising the ROI. The stability of the rate at which microsphere photons were detected during the data acquisition was then visually verified as a relatively even spread of points about their mean in a scatter plot of the microsphere photon counts determined for the set of images.

Given the assumption of a constant signal level and a constant background level throughout the acquisition of a data set, the mean microsphere photon count N_{photon} and the mean per-pixel background photon count β_0 are the same for each image in the data set. To obtain estimates of these two parameters, we added all the images in the data set to produce a sum image on which the estimation was carried out. The summing of images is desirable because it increases the amount of data on which estimation is performed, thereby improving the accuracy with which a parameter can be estimated. Specifically, using our microsphere image model and MATLAB's *lsqnonlin* function, a nonlinear least squares optimization was carried out on the same ROI that was used for the verification of signal and background stability from the individual images. In the microsphere image model, the radius r was set to half the average diameter specified by the manufacturer for the particular microsphere sample, or to half the nominal diameter if the average diameter for the sample was different by no more than 2%. The quantities estimated consisted of the x_0 and y_0 coordinates of the microsphere, the term $\frac{2\pi n_a}{\lambda}$ of Eq. (5), and the parameters N_{photon} and β_0. Since the values obtained for N_{photon} and β_0 pertained to the sum image, they were each divided by the number of images in the data set, and the results were used as fixed values for N_{photon} and β_0 in the maximum likelihood localization carried out on each individual image of the data set.

For the calculation of the limits of the localization accuracy to which the obtained localization accuracies were compared, the positional coordinates x_0 and y_0 were given by the mean of the x_0 estimates and the mean of the y_0 estimates, respectively, since the true values are not known for the experimental data sets. The radius r was set to either half the sample's average diameter or half the nominal diameter as described above, and the term $\frac{2\pi n_a}{\lambda}$ was set to the value determined from the sum image by the nonlinear least squares estimation.
